# Biomimetic Pseudopeptides to Decipher the Interplay between Cu and Methionine‐Rich Domains in Proteins

**DOI:** 10.1002/chem.202403896

**Published:** 2025-01-09

**Authors:** Joel I. Badillo‐Gómez, Irene Suarez‐Antuña, Ievgen Mazurenko, Frédéric Biaso, Jacques Pécaut, Elisabeth Lojou, Pascale Delangle, Sarah Hostachy

**Affiliations:** ^1^ Univ. Grenoble Alpes CEA, CNRS, Grenoble INP, IRIG, SyMMES 38000 Grenoble France; ^2^ Aix Marseille Univ CNRS, Laboratoire de Bioénergétique et Ingénierie des Protéines, Institut de Microbiologie de la Méditerranée 31 Chemin Aiguier 13402 Marseille France

**Keywords:** Bioinorganic chemistry, Copper binding, Methionine, Affinity, Tripodal ligand

## Abstract

Maintaining tightly copper homeostasis is crucial for the survival of all living organisms, in particular microorganisms like bacteria. They have evolved a number of proteins to capture, transport and deliver Cu(I), while avoiding Fenton‐like reactions. Some Cu proteins exhibit methionine‐rich (Met‐rich) domains, whose role remains elusive. In this work, we designed biomimetic compounds recapitulating the possible Cu(I) binding sites in these domains, in order to examine the parameters important for Cu(I) binding. Five different biomimetic pseudopeptides were synthesized, exhibiting either three methionines or two methionines and a third amino acid likely to be present in the Met‐rich domain. The affinities for Cu(I) of these model binding sites were determined, as well as their redox properties and behavior in the presence of Cu(II). Our results highlight the importance of Met residues, and their abundance in Met‐rich domains, to efficiently bind Cu(I) in the periplasmic space.

## Graphical abstract

Methionine‐rich domains, unity makes strength! Tripodal pseudopeptides mimicking individual binding sites in Met‐rich domains show that the trismethionine environment is highly favorable to stabilize the Cu(I) state. The moderate affinity of a single site is however amplified by efficient statistical and dynamic binding by the many methionine residues.

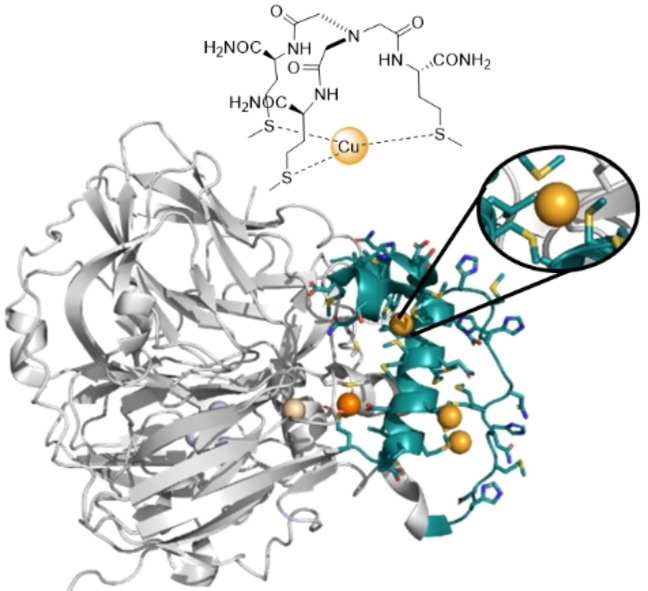

## Introduction

In all life kingdoms, copper is an essential element that performs key biological processes, often involving electron transfer and redox reactions. However, high concentrations of copper are toxic for the cells, for instance by generating the highly reactive hydroxyl radical through Fenton‐like reactions.[^1^, ^2^, ^3^, ^4^] For example, macrophages can induce high copper concentrations in their phagosomes to generate oxidative stress and kill entrapped pathogens.[^5^, ^6^] Like other organisms, bacteria have thus evolved mechanisms to tightly control copper concentrations and prevent its accumulation. A relevant example is the copper efflux system (Cue), which is one of the copper tolerance mechanisms in Gram‐negative bacteria like *E.coli*.[^5^, ^7^, ^8^] It is composed of a copper‐responsive metalloregulatory protein (CueR) that controls the expression of *copA* and *cueO* genes. CopA is a Cu(I)‐ATPase that transports Cu(I) from the cytoplasm to the periplasm. CueO, a periplasmic protein of the multicopper oxidase (MCO) family, oxidizes Cu(I)–Cu(II) while reducing oxygen to water. MCOs can also catalyze the oxidation of a number of phenolic substrates. Like other MCOs, CueO exhibits a type 1 (T1) copper center, where substrate oxidation occurs, and a trinuclear center (TNC), composed of one type 2 (T2) and two type 3 (T3) copper ions, where four‐electron oxygen reduction occurs.^[8]^ Unlike other MCOs, however, CueO possesses an additional methionine‐rich domain (Met‐rich) that hinders the access to the T1 center and provides additional Cu‐binding sites.^[9]^ For instance, crystal structures of *E.coli* CueO show three additional copper sites on this domain.[^10^, ^11^, ^12^] Cu5, also referred to as regulatory copper (rCu) or substrate copper (sCu), is located near the T1‐site and bound to two aspartate (Asp) and two methionine (Met) residues, while Cu6 and Cu7 are more solvent‐exposed and bound solely to Met residues. The exact role of these copper sites, and more specifically the contribution of this Met‐rich domain to CueO function in the context of copper resistance are still unclear.

Affinity is one of the key parameters to understand metal‐protein interactions. Met‐rich domains are known to preferentially bind Cu(I) because of the soft character of the thioether functions of Met side‐chains. The binding interaction of Met‐rich domains for Cu(I) is expected to remain low with respect to cysteine‐rich binding sites found in many Cu transporters.^[13]^ Met‐rich domains like the CueO one may possess several copper sites and numerous copper‐binding residues that could provide alternative or additional binding sites. However, the influence of the exact nature of the binding site involving several Met but also other amino acid side‐chains is poorly described. Considering the large number of potential binding motifs, and the flexibility of Met‐rich domains, determining the affinity of a given binding site for copper is challenging on the whole protein.

Biomimetic complexes that reproduce the metal coordination environment are attractive tools to model isolated metal‐binding sites in proteins and determine the binding parameters through easier and more accurate approaches. Several biomimetic strategies allowing to model Cu(I) Met‐rich sites have been reported in the literature. The pioneering work of Franz et al. demonstrated that Met‐rich linear peptides, which sequences are extracted from the copper transport protein Ctr1, bind Cu(I) through three Met residues in a Cu(SMet)_3_ coordination, with binding constant values (log K) between 5 and 6.[^14^, ^15^, ^16^, ^17^] Another biomimetic approach developed in our group consists in the pre‐orientation of several chosen amino acids side chains towards the metal center in well‐defined peptide‐based structures. The careful design of constrained cyclic decapeptides and tripodal pseudopeptides based on a chemical scaffold enables the organization of the metal coordinating moieties in space and affords metal complexes of larger stability than unstructured model peptides. The structural constrains imposed in these small model compounds roughly mimic the overall constrains of the whole protein tridimensional structure. This approach was successful in designing relevant mimics of high affinity Cu(I) thiolate binding sites found in copper transporters.[^18^, ^19^, ^20^, ^21^] Examining the resulting complexes in terms of stoichiometry, affinity or coordination mode provided useful insights into metal‐protein interactions. This strategy was extended to trismethionine binding sites as models of the N‐terminus of the Ctr1 transporter or to trishistidine coordination to get a pseudopeptide able to reasonably compete with the Aβ peptide for Cu.[^22^, ^23^]

Here, we propose to use this strategy in order to investigate the copper‐binding properties of protein Met‐rich domains. We designed and synthesized a series of biomimetic ligands reproducing known or putative Cu binding sites within the Met‐rich domain. We investigated Cu(I) binding first, since Cu(I) is known to be preferentially bound to the soft Met‐rich binding sites. We further demonstrated that Cu(II) binding was even weaker except for the pseudopeptide having a Met replaced by a His. The pseudopeptides presented in this paper give information about isolated Cu(I) binding sites found in Met‐rich domains with various coordination involving Met residues. The influence of the third amino acid on the coordination is discussed with a focus on the impact on the biological function of the Met‐rich domains.

## Results

### Design of Met‐Rich Domains Cu Binding Site Mimics

**Figure 1:**
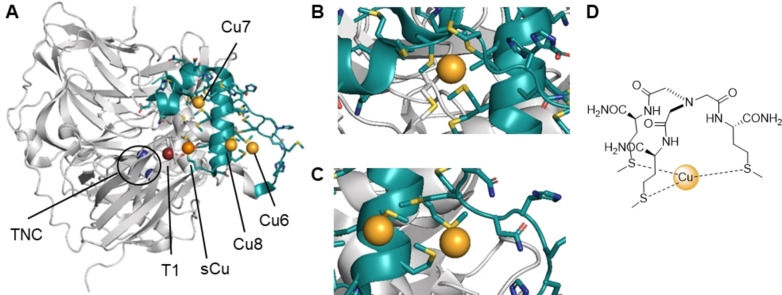
Design of pseudopeptide mimics of *Ec*CueO Cu(I) binding sites. (A) Ribbon model of *Ec*CueO C500S mutant (PDB: 3NT0), highlighting the Met‐rich domain (M355‐G399, teal) and Cu sites: TNC (dark blue), sCu (orange), and Met‐rich associated Cu6, Cu7, Cu8 (gold). Additional copper centers outside the Met‐rich domain and the canonical sites are not represented. For illustration purposes, the T1 site, absent from the mutant structure, is shown in dark red. Likewise, part of the Met‐rich domain is unresolved in the C500S mutant structure, but is represented in the figure by superimposing the WT structure (PDB: 3OD3). (B) Zoom on Cu7, coordinated by 3 Met residues. (C) Zoom on Cu6 and Cu8, where only part of the coordination sphere was resolved. (D) Proposed biomimetic model of the trismethionine binding sites.

We sought to reproduce the structure of potential Cu(I) binding sites in Met‐rich domains using a peptidomimetic approach, where amino acid side chains important for copper coordination are spatially arranged in a way that mimics the binding site of the protein. As a starting point, we examined the structure of wild‐type (WT) *E. coli* CueO (PBD: 3OD3) and a Cys500Ser (C500S) mutant missing the T1 site (Figure 1A). This C500S mutant is particularly interesting for our study, since its crystallographic structure reveals several additional Cu(I)‐binding sites in the Met‐rich region (PDB: 3NT0, Cu6–8, Figure 1A–C).^[12]^ In the C500S mutant structure, part of the Met‐rich region was unresolved, and extrapolated from the WT structure. The so‐called Cu7 site exhibited a trigonal Cu(I) bound to three Met with a trigonal planar geometry (Figure 1B). Other binding sites present in the Met‐rich domain only exhibited one or two coordinating Met, with a geometry suggesting non‐resolved additional ligands (Figure 1C). Tripods based on a nitrilotriacetic acid scaffold were previously used for mimicking the trigonal planar geometry of Cu(I) biological binding sites containing three identical residues Met, Cys or His.[^18^, ^19^, ^20^, ^22^, ^23^, ^24^] We thus reasoned that we could use a similar strategy to mimic the trigonal planar binding of the Cu(I) center expected in Met‐rich domains through Met and other proximal amino acids (Figure 1D). In these structures, only the side‐chains of the amino acids are binding Cu(I) with no involvement of the distant apical nitrogen, by contrast to tripodal chelates with similar donor atoms.^[25]^ Therefore, the pseudopeptides proposed here are reminiscent of the binding sites found in proteins.

**Scheme 1:**
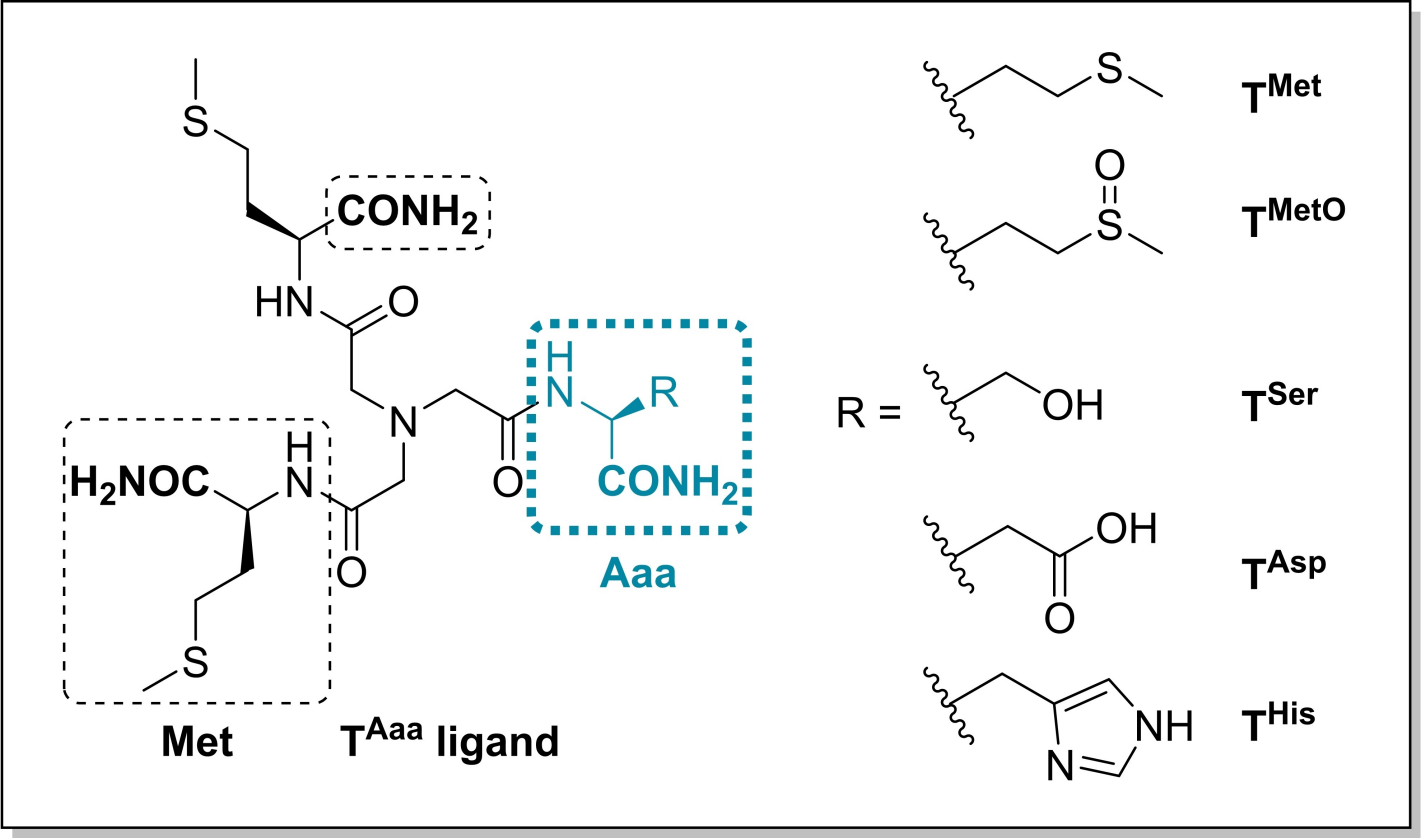
Series of tripodal pseudopeptides developed in this paper.

We thus decided to design and synthesize Met‐rich tripods bearing two Met residues and a third amino acid that could participate in Cu(I) coordination in the binding sites showing two Met only and an extra unknown ligand. Besides Met residues, the unresolved loop of the Met‐rich domain of *Ec*CueO C500S contains several histidines (His) as well as aspartic acids (Asp) and non‐coordinating residues such as glutamine (Gln), serine (Ser) or glycine (Gly). We thus decided to incorporate Met, His and Asp as the third residue to form **T^Met^**, **T^His^** and **T^Asp^** tripodal structures, respectively. In addition, Met oxidation raises an increasing interest since it is nowadays recognized to be involved in many biological processes, having different roles from protein damage, gene expression and enzymatic activity modulation.^[26]^ An additional question to be addressed is whether the elevated number of Met residues in CueO could protect Met from oxidation. A tripod incorporating an oxidized Met, **T^MetO^**, was thus added to the tripod series. Finally, Ser being a poor ligand of Cu(I), a **T^Ser^** tripod was also designed as a potential mimic for linear Cu(I) binding through two Met, only. The series of targeted Cu(I)‐binding tripods are shown in Scheme 1. The carboxylic acid of natural amino acids was replaced by an amide moiety to properly mimic the protein main chain. Besides, the presence of acidic groups in the tripodal ligands was demonstrated to be deleterious for Cu(I) coordination due to electrostatic repulsions between the three carboxylate anions at physiological pH.^[19]^

### Synthesis of Biomimetic Tripod Ligands

#### Synthesis of H‐Aaa‐NH_2_ Building Blocks

**Scheme 2:**
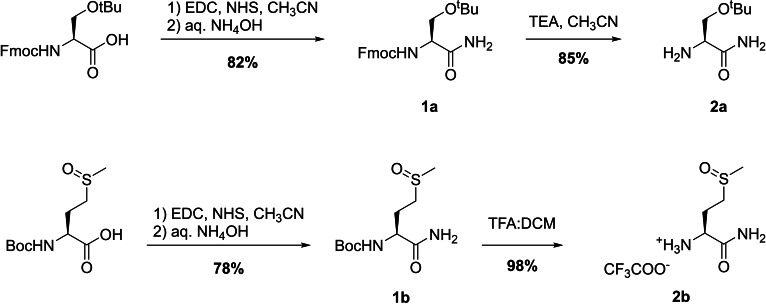
Synthesis of *H*‐Ser(tBu)‐*NH_2_* and *H*‐Met(O)‐*NH_2_* building blocks.

We first synthesized *H*‐Aaa‐*NH_2_* building blocks for all residues but Met, for which H‐Met‐NH_2_ was commercially available (Scheme 2). We used a similar strategy to the one previously developed in our group for d‐Penicillamine derivatives.^[27]^

We started from building blocks commonly used in solid‐phase peptide synthesis (SPPS), namely Boc‐Met(O)‐OH, Fmoc‐Asp(*t*Bu)‐OH, Fmoc‐Ser(*t*Bu)‐OH and Fmoc‐His(Trt)‐OH. The carboxylic acid was first activated using *N*‐(3‐(dimethylaminopropyl)‐N′‐ethylcarbodiimide (EDC) and N‐hydroxysuccinimide (NHS), followed by addition of an aqueous solution of NH_4_OH to form the desired amide **1** in good yields (70–82 %, see SI for detailed procedures). The conversion of the carboxylic acid to the amide was confirmed by the shift of α and β protons NMR signals and by mass spectrometry. All compounds were used without further purification.

The amine moieties were finally deprotected either with a 1 : 1 (v:v) mixture of TFA and DCM in the case of Boc‐Met(O)‐NH_2_, or an excess of triethylamine in CH_3_CN for the Fmoc‐protected compounds. Compounds **2** were obtained with good yields (83–98 %), and used without further purification in the following steps.

#### Synthesis of the Symmetrical Tripod

The symmetrical tripod bearing three Met units (**T^Met^**) was synthesized as previously reported for the ester derivative (see Supplementary Information).^[22]^ Briefly, nitrilotriacetic acid (NTA) was first converted to a NHS tri‐ester in presence of NHS. The resulting compound was then reacted with three equivalents of H‐Met‐NH_2_ in presence of N,N–diisopropylamine (DIPEA) in acetonitrile (ACN). **T^Met^** was obtained as a white solid in 57 % yield after purification by reverse‐phase preparative HPLC.

#### Synthesis of the Asymmetrical Tripods

**Scheme 3:**
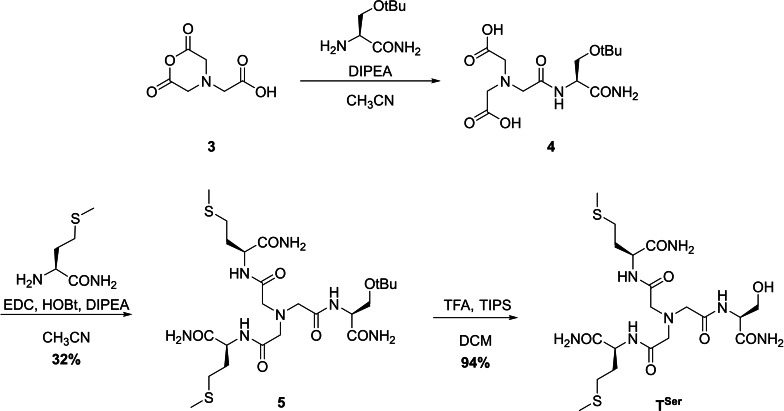
Synthesis of **T^Ser^** ligand.

A major objective was to break the symmetry of the NTA scaffold to obtain the four asymmetrical tripodal pseudopeptides **T^Asp^**, **T^His^**, **T^Ser^** and **T^MetO^**. In those cases, to enable the activation of only one carboxylic acid of the NTA core, the NTA anhydride **3** was formed in presence of acetic anhydride and pyridine.^[28]^ This compound was reacted with non‐Met building blocks in the presence of DIPEA in CH_3_CN to produce intermediates **4** (Scheme 3 shows the example of **T^Ser^**, see SI for additional experimental details and schemes). The crude compound was then reacted with *H*‐Met‐*NH_2_* in presence of EDC and 1–hydroxybenzotriazole (HOBt) to form **T^MetO^** or the protected tripods **5**. The MetO, Asp, and Ser derivatives were purified by reverse phase preparative HPLC to afford white solids in modest yields over two steps (14–32 %). The His derivative was not purified at this stage, but after the final deprotection. Finally, the protecting *t*Bu and Trt groups of Asp, His and Ser intermediates **5** were removed in presence of trifluoroacetic acid (TFA) and triisopropylsilane (TIPS) in DCM. All three tripods were purified by reverse phase preparative HPLC to afford white solids in modest global yields (13–30 %).

### Copper(I) Coordination Studies

**Figure 2:**
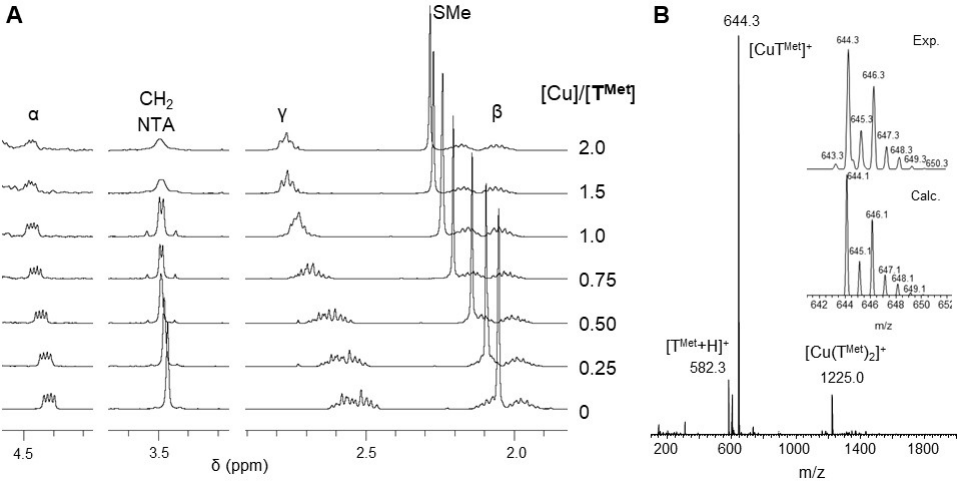
**A)** 400 MHz ^1^H NMR titration at 298 K in D_2_O of **T^Met^** (2 mM) with Cu(I), **B)** (+) ESI‐MS spectrum of Cu(I)**T^Met^** (200 μM) in ammonium acetate buffer (20 mM, pH 6.9), the inset shows the experimental (top) and calculated (bottom) isotopic envelops.

Having the five tripods in hand, we examined their Cu(I) coordination properties. Each ligand was stirred with one equivalent of [Cu^I^(CH_3_CN)_4_]PF_6_ for 3 h under Ar, and analyzed by electrospray ionization mass spectrometry (ESI‐MS). For all **T^Aaa^** ligands but **T^His^**, the signal of the mononuclear complex **Cu(I)T^Aaa^** was detected as the major species in the (+) ESI‐MS spectrum (Figure 2B and Figure S1). In addition to the signal of the mononuclear Cu complex, [Cu**T^Aaa^**]^+^, signals of lower intensity from the corresponding free ligand, [**T^Aaa^** +H]^+^ or [**T^Aaa^** +Na]^+^, and a small signal corresponding to **Cu(T^Aaa^)_2_** could also be observed. In the case of **T^His^**, the isotopic distribution for the signal at m/z=650.3 showed the superimposition of the signals due to both the Cu(I) and Cu(II) complexes, *i. e*. [Cu(I)**T^His^**]^+^ and [Cu(II)**T^His^**‐H]^+^. The presence of the Cu(II) species can be explained by the oxidation of the sample when getting out of the glovebox and the greater stability expected for this Cu(II) complex due to the His donor (see infra).

^1^H NMR experiments were carried out to corroborate the coordination of Cu(I). The affinity of the neutral tripods for Cu(I) is expected to be low. Therefore, to avoid possible competition with the CH_3_CN from the Cu(I) precursor,^[29]^ and to ensure complete coordination, we decided to use CuSO_4_ as the copper source and reduce it to Cu(I) *in situ* using dithionite (Na_2_S_2_O_4_). Increasing amounts of Cu (up to 2 eq.) were added to the ligand solutions, and the ^1^H NMR spectra of the resulting samples were recorded (Figures 2A and S2–5). *t*BuOH was added as a non‐coordinating, pH‐insensitive internal standard for signal chemical shift calibration.

Upon copper addition, and for all ligands, the spectra displayed a downfield displacement of the signals of the methyl and the γ protons of the two or three Met side chains that plateaued around 0.2 ppm above 1 equivalent of Cu(I). The α and β protons of Met residues also shifted, but to a lesser extent (0.04–0.07 ppm) (See Table 1, Figures 2 and S2–5). The signal of the methylene protons of the NTA scaffold (called CH_2_ NTA in Figure 2) evolves to an AB system upon Cu(I) coordination, which is characteristic of the greater rigidity found in the complex with respect to the free ligand. Altogether, this hints towards the coordination of Cu(I) by all Met residues of the ligands through the sulfur atoms of the thioether functions. In excess of Cu(I) all signals tend to broaden, possibly due to the formation of polymetallic species, which are very common in Cu(I) coordination chemistry.

**Table 1:** Characteristic data for the Cu**T** complexes.

Tripod	Δδ (ppm)^[a]^		logβ(Cu^I^ **T**)	E (V vs NHE)^[d]^	logβ(Cu^II^ **T**)^[f]^
	SCH_3_	Hγ			
**T^Met^**	0.19	0.19	8.18(8)^[b]^	0.33	4.8
**T^MetO^**	0.20	0.16	7.5(2)^[c]^	0.26	5.3
**T^Ser^**	0.21	0.19	7.6(2)^[c]^	0.28	5.1
**T^Asp^**	0.21	0.19	7.4(2)^[c]^	0.23	5.7
**T^His^**	0.21	0.16	8.81(15)^[b]^	n.d.^[e]^	≫9

^[a] 1^H NMR Shifts of the Met protons in the Cu(I) complex vs the free Ligand in D_2_O, pD 7, 298 K. ^[b]^ logβ(Cu^I^ **T**) determined through competition experiments with Fs, as a reference Cu(I) ligand by colorimetric titrations. The error on the last figures is given into brackets. ^[c]^ logβ(Cu^I^ **T**) determined through competition experiments with **T^Met^**, as a reference Cu(I) ligand by ^1^H NMR titrations following the chemical shit of the SCH_3_ protons of the **T^Met^** ligand. ^[d]^ Redox potential measured by SWV in MES buffer pH 5.5. ^[e]^ n.d., not determined. No redox activity in the studied potential range. ^[f]^ Stability constant from the Cu(II)**T** complex extrapolated from the redox potential (Equation (2)).

We next examined the chemical shifts of the third amino acid residue in the case of asymmetrical tripods, to determine whether they were involved in Cu(I) coordination. In the case of MetO and Ser residues, no signal displacement was observed, showing that MetO and Ser are not involved in Cu(I) coordination in **T^MetO^** and **T^Ser^**, respectively. For **T^Asp^**, we observed a small shift of the α and β proton signals of the aspartic acid residue (0.02–0.05 ppm), which may indicate some binding of Cu(I) by the carboxylate moiety. Finally, for **T^His^**, the imidazole of the histidine moiety presented large shift of 0.09 and 0.26 ppm for the H4 and H2 protons of the imidazole ring, respectively, showing a coordination of Cu(I) by the imidazole function of the His residue. In conclusion, **T^Met^** and **T^His^** bind Cu(I) through their three amino acid side chains, **T^Ser^** and **T^MetO^** bind Cu(I) through their two Met residues only, while **T^Asp^** shows an intermediate behavior with a possible involvement of the Asp carboxylate in addition to the two Met in Cu(I) coordination.

### Determination of Affinity Constants for Cu(I)

We then sought to determine the binding affinities of the five tripodal ligands for Cu(I), in aqueous solution and at equilibrium. Various probes for Cu(I) have been reported and used for the determination of affinity constants of other ligands in competition assays.^[30]^ We initially performed the competition assay with ferrozine (Fz) (Figure S6), a compound that has a lower affinity for Cu(I) (logβ_2_=15.1) than bathocuproine disulfonate (BCS) or 2,2′‐bicinchoninic acid (BCA), hence allowing to measure affinities for Cu(I) ranging from 10^10^–10^14^.[^30^, ^31^] This moderate affinity of Fz prevented the use of [Cu^I^(CH_3_CN)_4_]PF_6_ as a Cu(I) source, since competition of CH_3_CN with Fz was observed in our experimental conditions. Cu(I) was thus generated *in situ* by reducing Cu(II) sulfate in presence of sodium ascorbate and hydroxylamine in MOPS buffer (50 mM, pH 7.4) (Figure S6).^[30]^ Yet, in these conditions and regardless of the tripodal ligand, more than 90 % of Cu(I) was displaced from Cu**T^Aaa^** towards the [Cu(Fz)_2_]^3−^ complex, showing that the affinity range of Fz was too high and not suited for these ligands (Figure S6).

An alternative to Fz is ferene (Fs), that forms a [Cu(Fs)_2_]^3−^ absorbing at 484 nm (ϵ_Cu(Fs)2_^484 nm^=6700 M^−1^ cm^−1^).^[30]^ This complex has a logβ_2_ of 13.7 for Cu(I) and is suitable to measure logβ between 8 and 12. Each tripod ligand was thus reacted with 1 equiv. of *in situ‐*generated Cu(I) for 3 hours. After addition of 2 equivalents of Fs and four hours of reaction, we observed that 85 % of Cu(I) was withdrawn from the **CuT^Met^** complex. This gives a value of logβ 8.18±0.08, a very low affinity for Cu(I) (Table 1), which is very close to the affinity limit achievable using Fs as a Cu(I) competitor. 73 % of Cu(I) was withdrawn from the **CuT^His^** complex, resulting in a slightly higher affinity of **T^His^** for Cu(I) than **T^Met^** (logβ=8.81±0.15). For all other ligands, more than 90 % of Cu(I) was displaced from the **CuT^Aaa^** by Fs, preventing the accurate determination of logβ and confirming the lower affinity for Cu(I) of these three ligands.

In the absence of available probes with lower affinity for Cu(I) than Fs, we designed an experiment to estimate a logβ value for **T^MetO^**, **T^Asp^**, and **T^Ser^** taking **T^Met^** as a reference ligand. Having in mind that, upon Cu(I) addition, the NMR signal of the methyl groups of Met residues would gradually and significantly shift downfield (up to 0.2 ppm, Figure 2), we reasoned that we could use this chemical shift as an indicator of the concentration of **CuT^Met^** in solution (Equation 1).^[32]^

(1) $$\delta^{exp}=\;\frac{\left[T^{Met}\right]\times \delta^{T^{Met}}+\left[CuT^{Met}\right]\times \delta^{{CuT}^{Met}}}{{\left[T^{Met}\right]}_{tot}}$$

**Figure 3:**
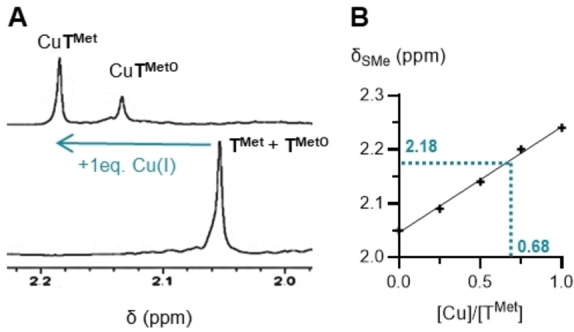
Competition of **T^Met^** and **T^MetO^** for Cu(I) followed by registering the SMe chemical shifts by ^1^H NMR. (A) SMe region of the ^1^H NMR spectra (400 MHz, 298 K, D_2_O) of an equimolar solution of **T^Met^** and **T^MetO^** (2 mM) in the absence (bottom) or in the presence (top) of 1 equiv. Cu(I). (B) The chemical shift of the SMe protons of **T^Met^** (δ_SMe_) varies linearly with [Cu(I)**T^Met^**] (see experiments Figure 2), thereby enabling to determine [Cu(I)**T^Met^**] from the δ_SMe_ value in the competition experiment (teal dotted lines).

In a competition experiment between **T^Met^** and **T^Aaa^** (Aaa=Asp, Ser, MetO), the Met methyl signals could be distinguished by their integration (9 protons for **T^Met^**, 6 for T^Aaa^). We would thus be able to determine the partition of Cu(I) between both ligands and to deduce the binding constants for **T^Aaa^**. We thus prepared a solution with stoichiometric amounts of **T^Met^** and **T^Aaa^** in D_2_O, and *t*BuOH as a reference. Cu(I) was then generated *in situ* by addition of CuSO_4_ and dithionite to the mixture. After 3 hours, a ^1^H NMR spectrum was recorded, and we examined the NMR signals of the final methyl groups of Met residues. As expected, the methyl signals of **CuT^Met^/T^Met^** and **CuT^MetO^/T^MetO^** could be easily identified thanks to their distinct chemical shifts and integration, and we could determine the concentrations of all species in the NMR samples (Figure 3). The calculated log β for all three ligands were comprised between 7.4 and 7.6 (Table 1).

### Cu(II) Coordination Studies

**Figure 4:**
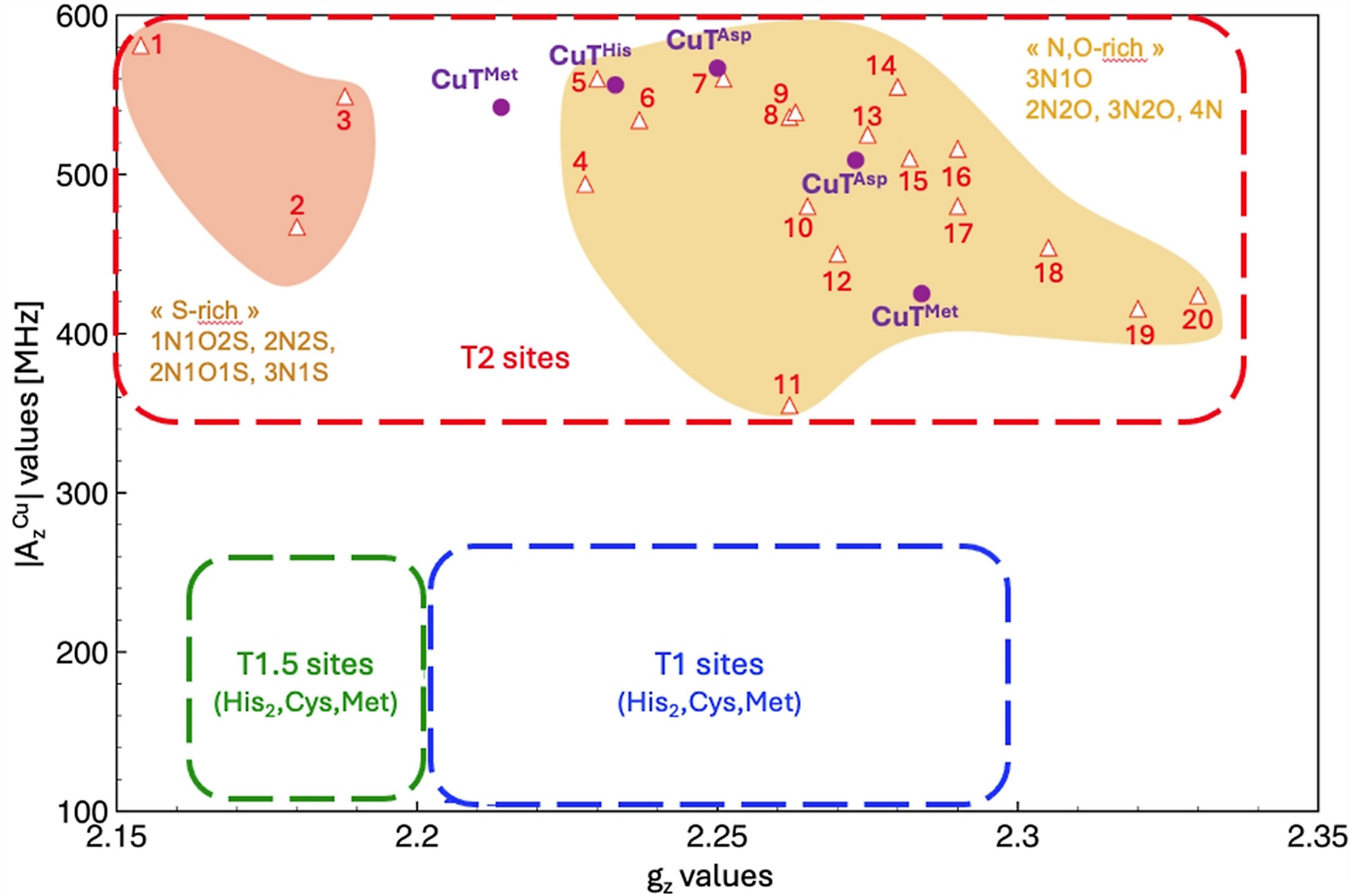
Peisach‐Blumberg diagram for Cu(II) protein sites. (1) *Bs*Sco_eq_,^[36]^ (2) HCC‐*Bs*Sco,^[37]^ (3) *Bs*Sco C45 A,^[38]^ (4) amyloid beta high pH,^[39]^ (5) *Pl*AA10 high pH,^[34]^ (6) *Rv* laccase,^[40]^ (7) *Bs*Sco_tr_,^[36]^ (8) *Rg*CopI T2,^[41]^ (9) amyloid beta low pH,^[42]^ (10) erythrocuprein,^[43]^ (11) *Pl*AA10 low pH,^[34]^ (12) benzylamine oxidase,^[44]^ (13) *Jl* hemocyanin,^[45]^ (14) Galactose oxidase,^[46]^ (15) dopamine‐β‐hydroxylase,^[47]^ (16) BS Amine oxidase,^[48]^ (17) diamine oxidase,^[49]^ (18) *Pd* N_2_OR,^[50]^ (19) *Ax* nitrite reductase,^[51]^ (20) *Ac* nitrite reductase T2.^[51]^ EPR parameters of the Cu(II) pseudopeptide complexes determined by X‐band EPR on frozen solutions are reported on the Peisach‐Blumberg diagram. The values obtained for Cu(II)**T^Met^**, Cu(II)**T^MetO^** and Cu(II)**T^Ser^** being almost identical, only Cu(II)**T^Met^** values are represented on the diagram.

CueO‐like proteins are found in the periplasm, where copper can be found both as Cu(I) and Cu(II). In addition, CueO has a cuprous oxidase activity, thereby generating Cu(II). The interaction of Cu(II) was therefore investigated with the five tripodal ligands. The EPR spectra of 100 μM solutions of CuSO_4_ and an excess of the ligands in acetate buffer (50 mM, pH 5) clearly indicate the presence of significant amounts of Cu(II) interacting with the buffer. This result is in line with the expected low affinity of the Met‐based ligands for the borderline cation Cu(II). Therefore, new experiments were conducted in a non‐competing buffer, *i. e*. MES (50 mM, pH 5.5). The spectra obtained for the Cu(II) complexes with the three ligands **T^Met^**, **T^MetO^** and **T^Ser^** are almost identical and indicate the presence of two different Cu(II) species that differ only in their ratio (Figure S7). Two other species are detected for the Cu(II)**T^Asp^** complex whereas only one Cu(II) species is detected with the fifth ligand bearing a histidine, **T^His^** (Figure S7). The characteristic values (g_z_ and A_z_^Cu^) are presented in Table S1 and plotted on a so‐called Peisach‐Blumberg diagram^[33]^ to get some insights about the atoms coordinated to Cu(II) in these complexes (Figure 4). For all studied complexes, signals fall within the region corresponding to the T2 protein sites. In the case of Cu(II)**T^Asp^**, both signals are located in the middle of this region where the known copper sites are characterized by a tetra‐ or pentacoordination involving almost always two nitrogen and one or two oxygen atoms bound to Cu(II). Such a coordination can be achieved thanks to the many oxygen and nitrogen donors available in the ligands: the carbonyl and the amidate functions due to the deprotonation of some of the secondary amide groups in the amino acid arms, as well as the carboxylate from the Asp arm. For Cu(II)**T^His^**, the EPR parameters closely match those reported for the *Pl* AA10 enzyme at pH 8.5 where Cu(II) is proposed to be coordinated by three nitrogen atoms and one oxygen atom.^[34]^ The well‐known strong affinity of imidazole rings to Cu(II) suggests that the His arm is likely coordinated by one nitrogen atom. Finally, for Cu(II)**T^Met^**, Cu(II)**T^MetO^** and Cu(II)**T^Ser^** complexes, the two EPR‐detected species belong to different part of the Peisach‐Blumberg diagram. The signal with the highest g_z_ value (g_z_=2.284) is located in the same region as some pentacoordinated Cu(II) sites with three nitrogen and two oxygen atoms bound to the metal. The other signal has a significantly lower g_z_ value (g_z_=2.214) that could arise from the presence of one or two sulfur atoms coordinated to Cu(II). For this complex, we could suggest coordination by one or two methionine arms. Interestingly, this signal matches with one of those observed recently in the protein, i. e. *Ec*CueO.^[35]^

**Figure 5:**
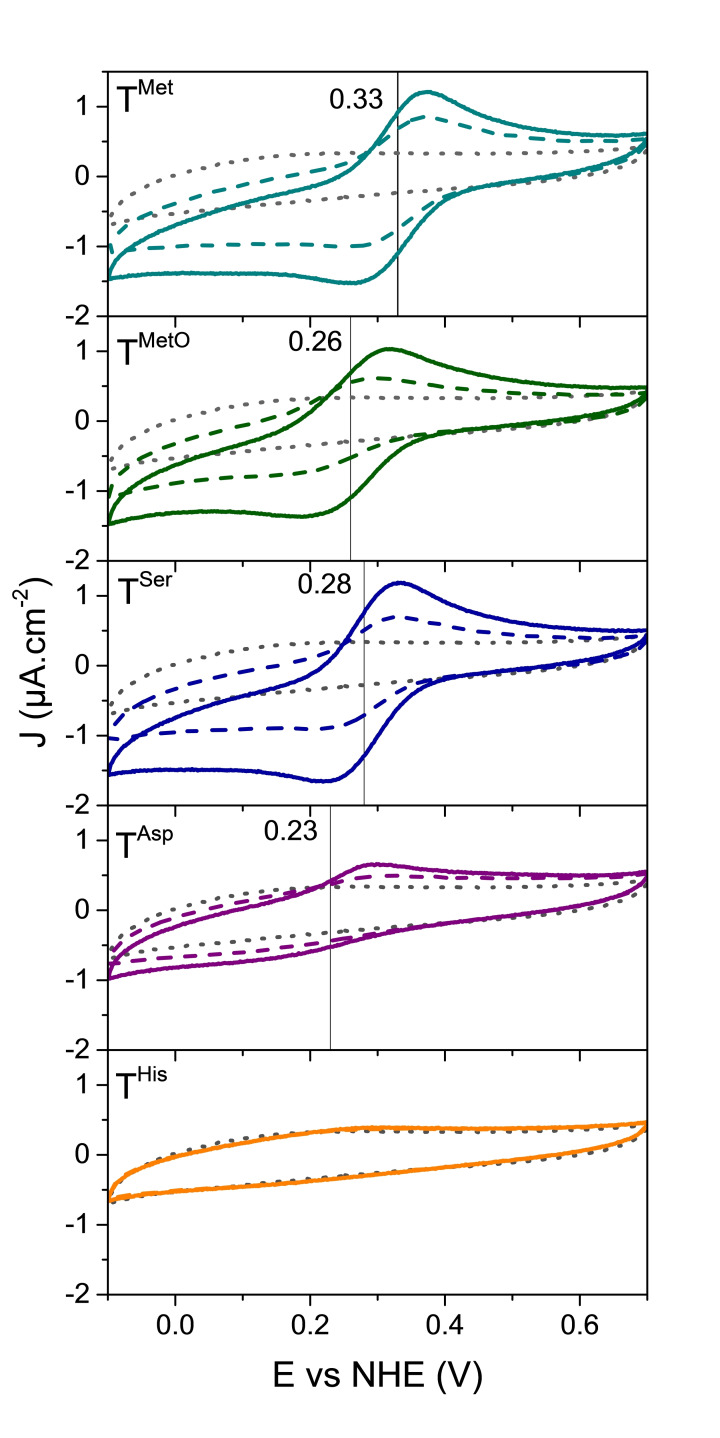
CVs of solutions of **T^Met^**, **T^MetO^**, **T^Ser^**, **T^Asp^** and **T^His^** (1 mM) in MES buffer (50 mM, pH 5.5). Scan rate: 2 mV s^−1^. [CuSO_4_]=0 μM (black dotted curve), 20 μM (colored dashed curve), 50 μM (colored plain curve). The formal potentials of the complexes are indicated on the CVs as vertical lines, as determined with square‐wave voltammetry (SWV) experiments on the same samples (see Figure S8). Blank experiments without tripods are shown in SI (Figures S9 and S10).

The redox properties of the series of Cu(II) complexes were then investigated by cyclic voltammetry (CV) in the same conditions as for EPR *i. e*. in MES buffer (50 mM, pH 5.5). The ligand concentration was fixed to 1 mM and CuSO_4_ was added to the medium until 50 μM. **T^His^** showed a peculiar behavior with no reduction of Cu(II)**T^His^** observed in the studied potential range, which suggests a sufficiently large stability for this Cu(II) species to prevent the formation of Cu(I)**T^His^** during the experiment. The four other tripods gave CVs exemplifying quasi reversible redox processes accounting for the formation of the Cu(I) complex by reduction of the Cu(II) species at the electrode (Figure 5). The value of the redox potential is significative of the relative stability of Cu(I)**T^Aaa^** and Cu(II)**T^Aaa^**. For example, the redox potential is significantly higher for **T^Met^** than for **T^MetO^**, which is consistent with a better stabilization of the Cu(I)**T^Met^** complex.

The redox potentials could be precisely measured for these four ligands thank to square‐wave voltammetry (SWV) experiments on the same samples (see Figure S8). Their values are reported in Table 1 and follow this order: **T^Met^** >**T^Ser^** >**T^MetO^** >**T^Asp^**. This trend in the redox potential values is correlated to a similar trend in the relative stabilities of the Cu(I) and Cu(II) complexes, β(Cu(I)**T**)/β(Cu(II)**T**), established for reversible systems (see SI).^[31]^ Therefore the softest sulfur‐rich ligand **T^Met^** exhibits the largest Cu(I) over Cu(II) selectivity whereas **T^Asp^**, which can promote the coordination of the hard aspartate group to Cu(II), exhibits the lowest Cu(I) over Cu(II) selectivity. The logβ(Cu(II)**T**) values evaluated from β(Cu(I)**T**) and the redox potential are given in Table 1.

The behavior of the complexes was also studied in the presence of oxygen by cyclic voltammetry to determine the stability of the Cu(I) complexes with **T^Met^**, **T^Ser^**, **T^MetO^** and **T^Asp^** towards oxidation. As seen on the CVs presented in Figure S11, with all the complexes except Cu(I)**T^Met^**, the appearance of the characteristic oxygen reduction wave at the tripod reduction peak and disappearance of the respective oxidation peak is significative of oxidation of the Cu(I) tripod complexes by oxygen. In the case of Cu(I)**T^Met^**, the reduction of oxygen occurs at a potential lower than the reduction of the tripod, showing a larger stability to oxidation of the Cu(I)**T^Met^** complex than the other studied tripodal ligands.

## Discussion and Conclusions

A series of five biomimetic ligands were designed and studied to model the expected binding sites for Cu(I) in Met‐rich domains found in some multicopper oxidases and copper transporters. Besides the predominant Met residue, His and Asp are often found in these domains and in the vicinity of Cu binding sites in X‐ray structures. Therefore, we synthesized tripodal pseudopeptides appended with two Met and a third amino acid, being Met, Asp or His. The two residues Ser and MetO were added to the series to model a non‐interacting amino acid and the monooxidation product of the trismethionine binding site, respectively.

**Scheme 4:**
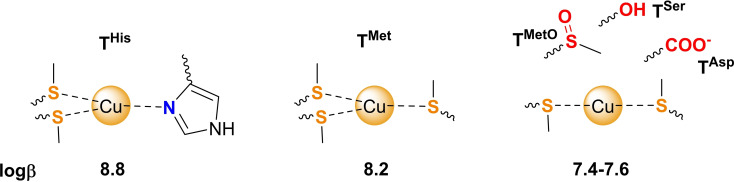
Proposed coordination modes for Cu(I) with the five tripodal ligands, with corresponding stability constants.

As expected, these biomimetic compounds with a soft character, adapted to Cu(I) coordination, form mononuclear Cu(I)**T** complexes exhibiting moderate affinity for Cu(I). The weak stability of the resulting Cu(I) complexes makes it challenging to determine the binding constants. We chose the lowest affinity probe proposed in the literature, *i. e*. ferene, to measure the affinity of two of the tripods, which display logβ slightly larger than 8. Then, the affinity of the three other ligands (logβ≈7.4–7.6) were determined by ^1^H NMR by following the proton shift of the SCH_3_ group, which is highly sensitive to Cu(I) binding. Indeed, ^1^H NMR was insightful for probing the chemical functions interacting with Cu(I) in the complexes thanks to proton chemical shits in the presence of Cu(I). As anticipated from the nature of the binding groups, the soft sulfur atoms of the thioether groups in Met are fully coordinated in each ligand, the borderline nitrogen atoms of His is also coordinated to Cu(I). By contrast, the absence of significant shifts of the side‐chains bearing hard donor groups in Ser, MetO and Asp strongly suggests their non‐involvement (or poor involvement) in the Cu(I) complex formation. Hence, we can propose the coordination modes drawn in Scheme 4 for the various Cu(I) complexes with coordination number CN=3 for **T^Met^** and **T^His^** vs CN=2 for **T^Ser^**, **T^MetO^** and **T^Asp^** with a probable digonal coordination. The two most stable binding sites are the triscoordinated ones with either three Met or two Met and one His. The three other sites display a slightly lower affinity for Cu(I), of less than one order of magnitude compared to **T^Met^**. Such a small difference in affinity between trigonal and digonal binding sites for Cu(I) is also found with peptides bearing negatively charged thiolate from cysteines, which exhibits logβ of 15–17 and 18–19 for digonal CuS_2_ and trigonal CuS_3_ coordination modes, respectively.[^19^, ^52^]

Interactions of the pseudopeptides with Cu(II) are even weaker, with the notable exception of **T^His^** and, to a lesser extent, **T^Asp^**. Comparison of the EPR data with the literature through Peisach diagram indicates that N/O binding atoms are preferred in the coordination sphere, but that some sulfur contribution may exist for some of the tripodal Cu(II) complexes. In the case of the [Cu(II)**T^His^**]^2+^ complex, the interaction of one borderline nitrogen of the imidazole ring with the borderline Cu(II) ion seems to strongly stabilize the Cu(II) state, since no reduction to Cu(I) could be observed by CV in MES buffer, contrarily to the other pseudopeptide tripods. It should be noted that these experiments were performed at a slightly acidic pH (pH 5.5) to prevent precipitation.

The biomimetic approach presented here provides insightful information about Cu binding to Met‐rich domains from proteins such as CueO‐like enzymes. First, the tris methionine pseudopeptide appears to provide one of the most favorable environments for coordinating Cu(I) in a CuS_3_ configuration. However, the stability of Cu(I)**T^Met^** is low as compared to other periplasmic proteins involved in copper homeostasis, e. g. CusF, a copper chaperone^[7]^ that helps exporting excess Cu(I) through the outer membrane via the CusABCD system (logK=9–14 for the protein or some model peptides,[^53^, ^54^, ^55^] Cu(I) interacting with a Met_2_HisTrp motif). This suggests that the Met‐rich domain is unlikely to function as a Cu(I)‐receptor or as a Cu(I)‐fixation site, as it would be exchanged readily with stronger protein binders. Nevertheless, while the affinity of a single Met‐site for Cu(I) might be low, the presence of multiple methionines and the flexible nature of the domain could enhance the affinity by contributing to Cu(I) binding with multiple Met side‐chains, increasing the probability to coordinate Cu(I). This hypothesis is supported by our recent study on *Ec*CueO, where we demonstrated that individual mutations of Cu6 or Cu7 Met‐sites do not affect the cuprous oxidase activity of the enzyme. However, deleting a substantial portion of the methionines or the entire Met‐rich domain does impact the activity, with the effect being more pronounced when a stronger Cu(I) complex is used as the substrate.^[35]^ This indirectly suggests that the Met‐rich domain may function more as a “copper sponge,” absorbing and transiently holding copper ions rather than harboring well‐defined, individual binding sites. This role would remind the function of Ctr proteins in yeast, in which series of clustered Met are involved in copper uptake.^[56]^ It should also be noted that, although the affinity of **T^Met^** is relatively low, it is still sufficient to provide some protection against Cu(I) oxidation by oxygen, unlike other tripods. This characteristic could be biologically significant, as it might help to prevent the formation of ROS, as a byproduct of the oxidation reaction.

The non‐involvement of Ser, Asp and MetO side chains in Cu(I) coordination, demonstrated in pseudopeptides **T^Ser^**, **T^Asp^** and **T^MetO^**, strongly suggests that these amino acids do not significantly contribute to Cu(I) binding in Met‐rich domains. In particular, the tripod **T^MetO^** illustrates the impact of Met oxidation, which occurs *in vivo*, on Cu binding. The oxidation of one Met residue in the tripods **T^MetO^** would result in a small decrease of affinity for Cu(I). Yet, the abundance of Met in the domain could provide alternative Met residues to replace oxidized ones in Cu(I) binding, thereby alleviating the consequences of Met oxidation.

However, the most striking differences are observed through replacement of one Met by a His, which significantly increases the affinity for Cu(I) and even more for Cu(II). His residues are found in combination with Met residues in many proteins involved in copper transport (as for example in CopC or CusF). Peptides modeling human Ctr1 were shown to present a high affinity for Cu(I) (log *K*=10.2±0.2 at pH 7.4) at His‐His sites along with an additional His or Met ligand.^[16]^ The cellular prion protein (PrP^C^) is involved in copper transport and possesses a MKHM motif in a non‐structured region, in which Met and His residues were found to bind Cu(I), the binding to His being pH dependent.^[57]^ It was also recently shown that a His‐Met‐rich tail in CopI, a cupredoxin protein from *R. gelatinosus*, was binding Cu(I) and required for Cu resistance.^[41]^ Therefore, His would be present to allow Cu(II) binding and to increase Cu(I) affinity with respect to pure Met binding sites as shown in the present biomimetic study comparing **T^His^** with **T^Met^**.[^58^, ^59^, ^60^] However, although 5 His residues are present in the Met‐rich domain of *Ec* CueO, no Cu(I) binding to these His sites could be identified.^[10]^ It can be hypothesized that the presence of numerous Met residues (14 for *Ec* CueO) in the Met‐rich domain with low Cu(I) binding affinity is preferable for the CueO function.

To conclude, the biomimetic pseudopeptides investigated in this work give insights into Cu binding to isolated sites found in Met‐rich domains from proteins such as CueO‐like enzymes and also into the contribution of other amino acids than Met to Cu binding in these domains. The trisMet site is undoubtedly a highly favorable coordination environment for Cu(I), which emphasizes the importance of Met residues to bind copper in the periplasm, a space submitted to O_2_. Oxidation of one Met to MetO in the trisMet binding site results in a decrease in affinity for Cu(I). However, the abundance of Met in the domain could balance the impact of Met oxidation. His is the only amino acid present in the domains that provides a higher affinity for Cu(I), albeit this affinity might be challenged at lower pH. His could also efficiently contribute to Cu(II) binding. The present biomimetic study together with results on the proteins strongly suggest that Met‐rich domains do not bind Cu in well‐defined, conserved individual Cu sites, but rather by statistical and dynamic binding by multiple Met, and possibly His, the overall affinity of this “Cu sponge” being much higher than the one of one single binding site.

## Author Contributions

All authors have reviewed the final manuscript.

JBG: investigation, resources, validation, visualization, writing‐original draft preparation; writing – review & editing; ISA: investigation, resources; IM: investigation, formal analysis, methodology, visualization, writing – review & editing; FB: investigation, formal analysis, methodology, visualization, writing – review & editing; JP: investigation, resources; EL: funding acquisition, conceptualization, project administration, writing – review & editing; PD: conceptualization, funding acquisition, project administration, formal analysis, validation, visualization, supervision, writing – original draft preparation, writing – review & editing; SH: conceptualization, formal analysis, funding acquisition, validation, visualization, supervision, writing – original draft preparation, writing – review & editing.

## Conflict of Interests

The authors declare no conflict of interest.

1

## Supporting information

As a service to our authors and readers, this journal provides supporting information supplied by the authors. Such materials are peer reviewed and may be re‐organized for online delivery, but are not copy‐edited or typeset. Technical support issues arising from supporting information (other than missing files) should be addressed to the authors.

Supporting Information

## Data Availability

The data that support the findings of this study are available from the corresponding author upon reasonable request.
