# Supplementary material for: Biomimetic Pseudopeptides to Decipher the Interplay between Cu and Methionine‐Rich Domains in Proteins
Source: Chemistry. 2025 Jan 9;31(11):e202403896. doi: 10.1002/chem.202403896 (PMC11840665; doi:10.1002/chem.202403896)
Supplement: Supplementary file 1 — Supporting Information [file CHEM-31-e202403896-s001.pdf]

Chemistry–A European Journal

Supporting Information

Biomimetic Pseudopeptides to Decipher the Interplay between Cu and Methionine-Rich Domains in Proteins

Joel I. Badillo-Gómez, Irene Suarez-Antuña, Ievgen Mazurenko, Frédéric Biaso, Jacques Pécaut, Elisabeth Lojou, Pascale Delangle,* and Sarah Hostachy*

Supporting Information

Biomimetic Pseudopeptides to Decipher the Interplay Between Cu and Methionine-Rich Domains in Proteins

Joel I. Badillo-Gómez,[a] Irene Suarez-Antuña,[a] Ievgen Mazurenko,[b] Frédéric Biaso,[b]

Jacques Pécaut,[a] Elisabeth Lojou,[b] Pascale Delangle,[a]* Sarah Hostachy[a]*

[a] Univ. Grenoble Alpes, CEA, CNRS, Grenoble INP, IRIG, SyMMES, 38000 Grenoble, France

[b] Aix Marseille Univ, CNRS, Laboratoire de Bioénergétique et Ingénierie des Protéines, Institut de Microbiologie de la Méditerranée, 31 chemin Aiguier, 13402 Marseille, France

Table of contents

1. Experimental procedures 3

General information........ 3

Abbreviations 3

Synthesis of H-Aaa(PG)-NH_2 building blocks. 4

Synthesis of the symmetrical tripod T^{Met} 9

Synthesis of the asymmetrical tripods 10

Physico-chemical studies. 17

2. Characterization of the tripodal ligands........ 19

3. Mass spectra of the Cu(I) complexes 29

4. NMR spectra of the Cu(I) complexes........ 30

5. Competition experiments 32

6. EPR spectra of the Cu(II) complexes........ 33

7. Electrochemistry of the Cu complexes 34

8. References........ 37

1. Experimental procedures

General information

Water solutions were prepared from ultrapure laboratory grade water that has been filtered and purified by reverse osmosis using Millipore MilliQ reverse-osmosis cartridge system (resistivity 18 MΩcm).

To prevent the oxidation of Cu(I), all solutions for physicochemical studies were prepared under an argon atmosphere.

Mass spectra were acquired on a LXQ-linear ion trap (THERMO Scientific) instrument equipped with an electrospray ion source. Electrospray full scan spectra have been recorded in the m/z = 50–2000 amu by infusion through a fused silica tubing at a flow rate of 2-10 mL/min. The temperature of the heated capillary for the LXQ was set in the range of 200-250°C, the ion-spray voltage was in the range of 3–6 kV and the injection time was 5-200 ms. The ligand solutions (≈ 200 µM) were prepared in ammonium acetate buffer (20 mM, pH 6.9).

The NMR experiments were recorded on a 400 MHz Bruker Avance spectrometer equipped with a BroadBand Inverse (BBI) probe with a z axis gradient field.

For hydrophilic final compounds, analytical HPLC were performed with a VWR system fitted with a Chromolith RP18 column (L = 100 mm, ∅ = 4.6 mm and p = 5 µm), with flow rates of 1 mL/min. Preparative HPLC were performed with a VWR system fitted with a Purosphere RP18 column (L = 250 mm, ∅ = 50 mm and p = 10 µm) with flow rates of 30 mL/min. Solvents: A : H_2O:TFA [99.9:0.1]. B : CH_3CN:H_2O:TFA [90:10:0.1].

NMR data

Abbreviations

Boc	tert-butyloxycarbonyl
DCM	Dichloromethane
DIPEA	*N,N*-diisopropylethylamine
EDC·HCl	*N*-(3-Dimethylaminopropyl)-*N'*-ethylcarbodiimide hydrochloride
Fmoc	Fluorenylmethoxycarbonyl
HOBt	1- Hydroxybenzotriazole
NHS	*N*-hydroxysuccinimide
NTA	Nitrilotriacetic acid
TEA	Triethylamine
TIPS	Triisopropylsilane

Synthesis of H-Aaa(PG)-NH_2 building blocks.

*General procedure A for the synthesis of Fmoc-Aaa(PG)-NH_2 or Boc-Met(O)-NH_2 (**1a-d**)*
N-hydroxysuccinimide (NHS) (2 equiv.) was added to a solution of Fmoc-Aaa(PG)-OH (1 equiv.) in anhydrous CH_3CN (11.5 mL/mmol) under argon. The solution was then cooled in an ice bath, and *N*-(3-Dimethylaminopropyl)-*N'*-ethylcarbodiimide hydrochloride (EDC·HCl) (2 equiv.) was added. The reaction mixture was allowed to reach room temperature overnight. After 18 h, the solvent was evaporated, the crude mixture was taken up in EtOAc and washed three times with saturated aqueous $NaHCO_3$. The organic layer was dried over Na_2SO_4, filtered and concentrated. The resulting crude was dissolved in anhydrous CH_3CN (15 mL/mmol) at room temperature. An aqueous ammonium hydroxide solution (28-30%, 8-9 equiv.) was added dropwise, and a white precipitate appeared. The reaction mixture was stirred for three hours at room temperature. Solvents were evaporated, the residue was taken up in hexane and washed three times with sat. aq. $NaHCO_3$.The organic layer was dried over Na_2SO_4, filtered and concentrated.

*General procedure B for the synthesis of H-Aaa(PG)-NH_2 from Fmoc-Aaa(PG)-NH_2 (**2a,c,d**)*
Compound **1** (1 equiv.) was dissolved in anhydrous CH_3CN (7.5 mL/mmol) under argon. Triethylamine (TEA) (40 equiv.) was added to this solution at room temperature. After 18 h, the solvent was evaporated, the residue was triturated with MeOH (5mL), resulting in a suspension. The white precipitate was filtered off and the filtered solution was evaporated. The resulting yellow oil was dissolved in a small quantity of MeOH and washed five times with hexane (15mL), until the Fmoc byproduct could no longer be seen by TLC in the MeOH layer (TLC: DCM/MeOH 80:20 v:v). Solvent was evaporated to yield the desired compound.

Synthesis of Fmoc-Ser(OtBu)-NH2

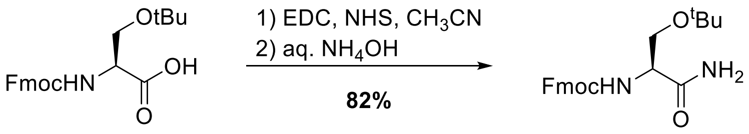

1a

Obtained using general procedure A, starting with Fmoc-Ser(OtBu)-OH (1 g, 2.6 mmol, 1 equiv.). Product obtained as a yellow oil (0.8164 g, 2.134 mmol, 82%).

^{1}H NMR (MeOD, 400 MHz, 298 K): δ (ppm) 7.81 (d, J=7.51 Hz, 2H, CH_{ar}), 7.67 (m, 2H, CH_{ar}), 7.41 (m, 2H, CH_{ar}), 7.33 (m, 2H, CH_{ar}), 4.43 (m, 2H, CH_2O), 4.25 (m, 2H, CH, H_α), 3.64 (m, 2H, H_β), 1.20 (s, 9H, CH_3).

^{13}C NMR (MeOD, 125 MHz, 298 K): δ (ppm) 174.19 (C=O(NH_2)), 156.94 (C=O(O)), 143.30 (C_{ar}), 141.21 (C_{ar}), 127.41 (CH_{ar}), 126.77 (CH_{ar}), 124.79 (CH_{ar}), 119.54 (CH_{ar}), 73.25 (C-CH_3), 66.64 (CH_2O), 61.72 (C_β), 55.37 (C_α, CH), 26.27 (CH_3).

ESI-MS (+): M = $C_{22}H_{26}N_2O_4$, m/z calculated for $[M+H]^+$ 383.20, found 383.2.

Synthesis of H-Ser(OtBu)-NH2

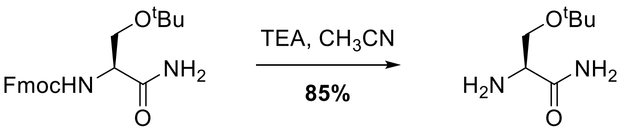

1a **2a**

Obtained using general procedure B, starting with compound **1a** (0.8164 g, 2.134 mmol). The product was obtained as a yellow oil (0.2918 g, 1.822 mmol, 85%).

^{1}H NMR (MeOD, 400 MHz, 298 K): δ (ppm) 3.63-3.53 (m, 3H, H_α, H_β), 1.22 (s, 9H, CH_3).

^{13}C NMR (MeOD, 125 MHz, 298 K): δ (ppm) 175.28(C=O), 73.16 (C-CH_3), 63.09 (C_β), 54.60 (C_α), 26.28 (CH_3).

ESI-MS (+): M = $C_7H_{16}N_2O_2$, *m/z* calculated for $[M+H]^+$ 161.13 ; obtained 161.1.

Synthesis of Fmoc-Asp(OtBu)-NH₂

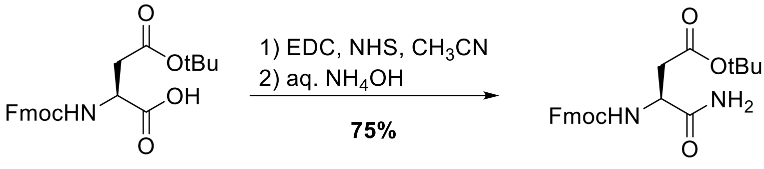

1c

Obtained using general procedure A, starting with Fmoc-Asp(OtBu)-OH (1 g, 2.430 mmol). Product obtained as a yellow oil ((0.7473 g, 1.821 mmol, 75%).

^{1}H NMR (MeOD, 400 MHz, 298 K): δ (ppm) 7.82 (m, 2H, CH_{ar}), 7.66 (m, 2H CH_{ar}), 7.40 (m, 2H, CH_{ar}), 7.32 (m, 2H, CH_{ar}), 4.52 (m, 1H, H_{α}), 4.42 (m, 2H, CH_2O), 4.24 (m, 1H, CH), 2.79 (dd, 16.03, 5.28 Hz, 1H, H_{β}), 2.58 (dd, 16.04, 8.57 Hz, 1H, H_{β}), 1.45 (s, 9H, CH_3).

^{13}C NMR (MeOD, 125 MHz, 298 K): δ (ppm) 174.60 (NH_2-C=O), 170.02 (O-C=O), 156.87 (NH-C=O(O)), 143.82 (C_{ar}), 141.22 (C_{ar}), 127.40 (CH_{ar}), 126.77 (CH_{ar}), 124.81 (CH_{ar}), 119.54 (CH_{ar}), 106.82 (C-CH_3), 80.99 (CH_2O), 66.65 (C_{β}), 51.55 (C_{α}), 37.33 (CH), 26.90 (CH_3).

ESI-MS (+): M = $C_{23}H_{26}N_2O_5$, *m/z* calculated for $[M+Na]^+$ 433.17, found 433.3.

Synthesis of H-Asp(OtBu)-NH₂

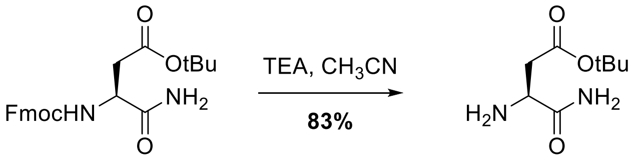

1c **2c**

Obtained using general procedure B, starting with compound **1c** (0.7473 g, 1.821 mmol). The product was obtained as a yellow oil (0.2842 g, 1.511 mmol, 83%).

^{1}H NMR (MeOD, 400 MHz, 298 K): δ (ppm) 3.69-3.66 (dd, J=12.52, 1.98 Hz, 1H, H_{α}), 2.70-2.58 (dd, J= 7.26, 5.26 Hz, 2H, H_{β}), 1.48 (s, 9H, CH_3).

^{13}C NMR (MeOD, 125 MHz, 298 K): δ (ppm) 177.21 (NH_2-C=O), 170.79 (O-C=O), 80.92 (C-CH_3), 51.26 (C_{β}), 39.81 (C_{α}), 26.92 (CH_3).

ESI-MS (+): M = $C_8H_{16}N_2O_3$, *m/z* calculated for $[M+H]^+$ 189.12, found 189.0.

*Synthesis of Fmoc-His(Trt)-NH$_2$ (**1d**)*

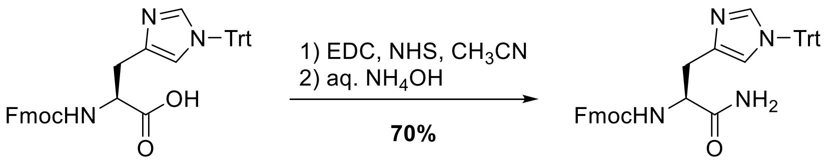

1d

Obtained using general procedure A, starting with Fmoc-His(Trt)-OH (1 g, 1.613 mmol). Product obtained as a yellow oil (0.6985 g, 1.129 mmol, 70%).

^{1}H NMR (MeOD, 400 MHz, 298 K): δ (ppm) 8.89 (s, 1H, $H_{imidazole}$), 8.18 (s, 1H, $H_{imidazole}$), 7.45-7.44 (m, 14H, H_{ar}), 7.22-7.20 (m, 8H, H_{ar}), 7.13 (s, 1H, H_{ar}), 4.23 (t, J= 6.67, 1H, CH), 4.18 (t, J= 6.70, 1H, H_{α}), 3.44-3.37 (m, 2H, CH_2O), 3.23-3.16 (m, 2H, H_{β}).

^{13}C NMR (MeOD, 125 MHz, 298 K): δ (ppm) 169.21 (C=O(NH_2)), 143.99 (C-Ph), 141.14 (C=O(O)), 138.02 ($C_{imidazole}$), 134.57 ($C_{imidazole}$), 129.46 (C_{ar}), 128.45 (C_{ar}), 128.19 (C_{ar}), 127.34 (C_{ar}), 126.63 (C_{ar}), 121.54 (C_{ar}), 118.10 (C_{ar}), 52.33 (C_{α}), 51.76 (CH), 27.74 (C_{β}), 26.31 (CH_2O).

ESI-MS (+): M = $C_7H_{16}N_2O_2$, *m/z* calculated for $[M+H]^+$ 619.27, found 619.2.

*Synthesis of H-His(Trt)-NH$_2$ (**2d**)*

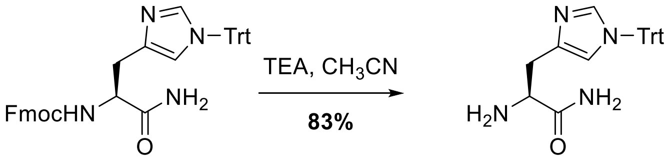

1d **2d**

Obtained using general procedure B, starting with compound **1d** (0.1900 g, 0.307mmol). The product was obtained as a yellow solid (0.1046 g, 0.264 mmol, 86%).

^{1}H NMR (MeOD, 400 MHz, 298 K): δ (ppm) 7.46 (s, 1H, $H_{imidazole}$), 7.45-7.38 (m, 9H, H_{ar}), 7.19-7.16 (m, 6H, H_{ar}), 6.82 (s, 1H, $H_{imidazole}$), 3.82-3.78 (t, J= 13.08 Hz, 1H, H_{α}), 3.02-3.83 (dd, J= 7.82, 5.70 Hz, 2H, H_{β}).

^{13}C NMR (MeOD, 125 MHz, 298 K): δ (ppm) 175.02 (C=O(NH_2)), 142.23 (C_{ar}), 138.46 ($CH_{imidazole}$), 135.73 ($C_{imidazole}$), 129.48 (C_{ar}), 127.87 (C_{ar}), 120.08 ($CH_{imidazole}$), 75.55 (C-Ph), 53.98 (C_{α}), 31.78 (C_{β}).

ESI-MS (+): M = $C_{25}H_{24}N_4O$, *m/z* calculated for $[M+H]^+$ 397.20, found 397.0.

Synthesis of Boc-Met(O)-NH_2

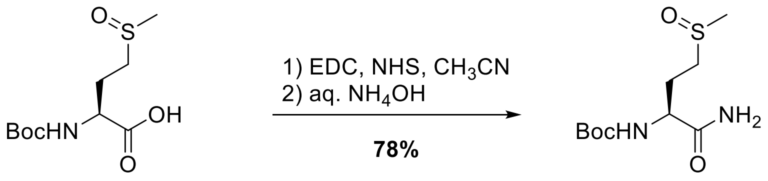

1b

Obtained using general procedure A, starting with Boc-Met(O)-OH (1 g, 3.768 mmol). Product obtained as a white solid (0.7762 g, 2.939 mmol, 78%).

^{1}H NMR (MeOD, 400 MHz, 298 K): δ (ppm) 4.24-4.19 (m, 1H, $H_α$), 2.96-2.81 (m, 2H, $H_γ$), 2.67 (s, 3H, S-CH_3), 2.27-2.18 (m, 1H, $H_β$), 2.10-1.98 (m, 1H, $H_β$), 1.47 (s, 3H, S-CH_3).

^{13}C NMR (MeOD, 125 MHz, 298 K): δ (ppm) 175.02 ((C=O(NH_2)), 156.34 (C=O(O)), 79.46 (O-C), 53.34 ($C_α$), 49.79 ($C_γ$), 36.76 (S-CH_3), 27.27 (C-CH_3), 25.23 ($C_β$).

ESI-MS (+): M = $C_{10}H_{20}N_2O_4S$, *m/z* calculated for $[M+H]^+$ 265.12, found 265.0.

Synthesis of H-Met(O)-NH_2

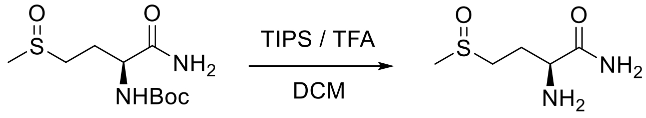

1b **98%** **2b**

In a round bottom flask and under an argon atmosphere, Boc-Met(O)-NH_2 (0.1745 g, 0.660 mmol) was dissolved in 4.5 mL of DCM, followed by the addition of TFA (4.55 mL, 59.463 mmol) to this solution. After this, TIPS (0.87 mL, 4.294 mmol) was added to the reaction was stirred for 2 h at room temperature. Then, the solvent was evaporated to dryness, and the mixture was triturated with Et_2O (3 × 5 mL). After separating the ether, the compound was dried under vacuum to afford a white solid (0.1062 g, 0.6468 mmol, 98 %).

^{1}H NMR (MeOD, 400 MHz, 298 K): δ (ppm) 4.11-4.07 (m, 1H, $H_α$), 3.11-3.01 (m, 1H, $H_β$), 2.94-2.86 (m, 1H, $H_β$), 2.71 (s, 3H, S-CH_3), 2.41-2.34 (m, 2H, $H_γ$).

^{13}C NMR (MeOD, 125 MHz, 298 K): δ (ppm) 169.90 (C=O), 51.73 ($C_α$), 36.53 (S-CH_3), 24.80 ($C_β$), 24.498 ($C_γ$).

ESI-MS (+): M = $C_5H_{12}N_2O_2S$, *m/z* calculated for $[M+H]^+$ 165.07, found 165.1.

Synthesis of the symmetrical tripod T^{Met}

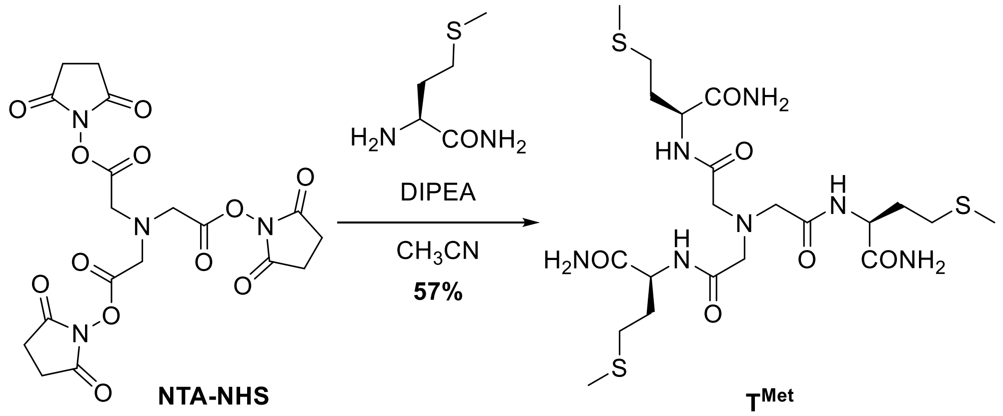

NTA-NHS was synthesized as previously reported.[1]

(*S*)-2-amino-4-(methylthio) butanamide hydrochloride (0.458 g, 2.48 mmol, 3 equiv.) was suspended in anhydrous CH_3CN (12 mL), followed by NTA-NHS (0.397 g, 0.823 mmol, 1 equiv.). DIPEA (1.3 mL, 7.47 mmol, 9.1 equiv.) was then added, yielding a transparent solution. The reaction mixture was stirred at room temperature for 24h, during which time a white precipitate formed. The reaction mixture was filtered through a fritted-glass filter, and the precipitate was washed with CH_3CN. The filtered solution was concentrated in vacuo to yield yellowish oil that was purified by reverse phase preparative HPLC. Gradient: 5% B for 2 min, 5 to 80% B in 15 min and 100% B for 2 min. After freeze-drying the product was obtained as a white solid (0.2773 g, 0.48 mmol, 57%). t_R = 8.4 min (analytical HPLC gradient: 5%B for 2 min, 5 to 100%B in 15 min, 100%B for 5 min).

^{1}H NMR (MeOD, 400 MHz, 298 K): δ (ppm) 4.53-450 (dd, J=14.09; 4.90 Hz, 3H, $H_{\alpha\text{-Met}}$), 3.66-3.55 (m, 6H, CH_2-N), 2.62-2.54 (m, 6H, $H_{\gamma\text{-Met}}$), 2.15-2.10 (m, 12H, $H_{\beta\text{-Met}}$, S-CH_3), 2.02-1.98 (m, 3H, $H_{\beta\text{-Met}}$).

^{13}C NMR (MeOD, 125 MHz, 298 K): δ (ppm) 175.19 (NH-C=O), 170.95 (NH_2-C=O_{Met}), 57.52 (CH_2-N), 52.47 ($C_{\alpha\text{-Met}}$), 31.23 ($C_{\beta\text{-Met}}$), 29.89 ($C_{\gamma\text{-Met}}$), 13.87 (S-CH_3).

ESI-MS (+): M = $C_{21}H_{39}N_7O_6S_3$, m/z calculated for $[M+H]^+$ 582.21, found 582.3.

Synthesis of the asymmetrical tripods

NTA anhydride **3** was synthesized following a reported procedure.[2]

*General procedure C for the synthesis of protected tripods (**5a-d**) and **T^{MetO}***

Compound **2** (1 equiv.) was dissolved in anhydrous CH_3CN (3.3 mL/mmol) under argon. DIPEA (2 equiv.) was then added, followed by NTA anhydride **3** (1 equiv.). The reaction mixture was stirred for 24 h at room temperature. CH_3CN was evaporated to afford a yellow oil that was used for the next step without purification. This oil was dissolved in anhydrous CH_3CN (40 mL/mmol) under an argon atmosphere. EDC·HCl (2.2 equiv.) and HOBt (2.3 equiv.) were then added to the reaction mixture, followed by DIPEA (6 equiv.) and H-Met-NH_2 (3 equiv.). The reaction was stirred at room temperature for 24 h, and the solvent was evaporated to yield a yellow oil. This crude oil was purified by reverse-phase preparative HPLC.

*Synthesis of **T**$^{Ser(tBu)}$ (5a)*

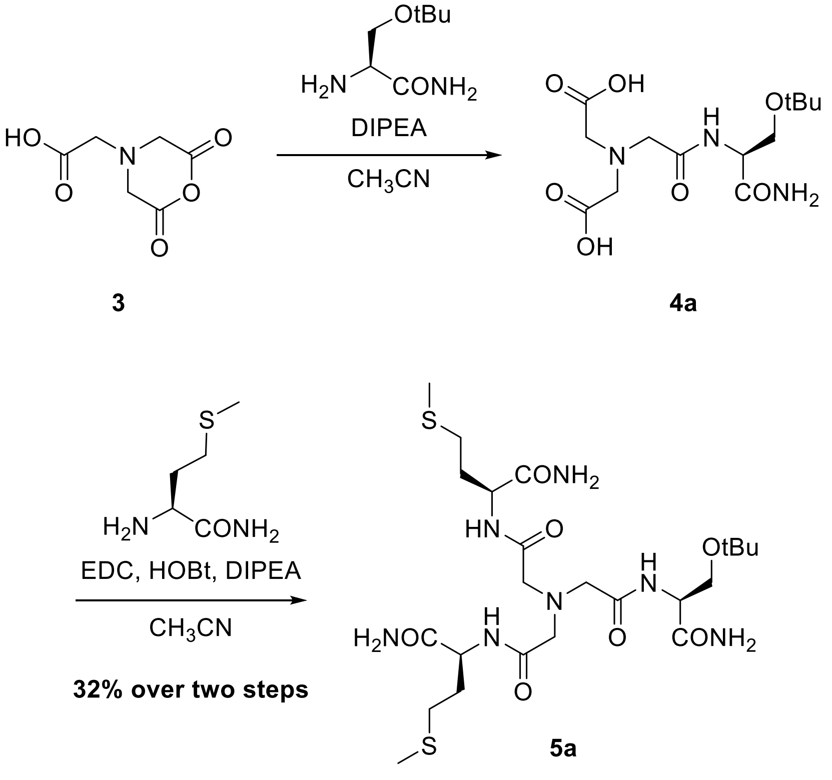

The compound was obtained following general procedure C starting from compound **2a** (50 mg, 0.312 mmol). HPLC gradient: 10% B for 2 min, 10 to 40% B in 18 min, 40% B for 2 min. The desired product was obtained as a white solid (0.060 g, 0.101 mmol, 32%). t_R = 10.38 min (analytical HPLC gradient 15%B for 2 min, 15 to 25%B in 15 min, 25%B for 2 min).

^{1}H NMR (MeOD, 400 MHz, 298 K): δ (ppm) 4.54-4.49 (m, 3H, $H_{α\text{-Ser}}$, $H_{α\text{-Met}}$), 3.79-3.73 (m, 2H, $H_{β\text{-Ser}}$), 3.69-3.62 (m, 6H, CH_2-N), 2.66-2.51 (m, 4H, $H_{γ\text{-Met}}$), 2.16-2.08 (m, 8H, $H_{β\text{-Met}}$, S-CH_3), 1.22 (s, 9H, C-CH_3).

^{13}C NMR (MeOD, 125 MHz, 298 K): δ (ppm) 175.20 (NH-C=O), 173.62 (NH_2-C=O_{Met}), 170 (NH_2-C=O_{Ser}), 73.37 (O-C), 61.44 (CH_2-N), 57.13 ($C_{β\text{-Ser}}$), 53.84 ($C_{α\text{-Ser}}$), 52.47 ($C_{α\text{-Met}}$), 31.26 ($C_{β\text{-Met}}$), 29.90 ($C_{γ\text{-Met}}$), 26.31 (CH_3), 13.86 (S-CH_3).

ESI-MS (+): M = $C_{23}H_{43}N_7O_7S_2$, m/z calculated for $[M+H]^+$ 594.27, found 594.3.

*Synthesis of **T^{Ser}***

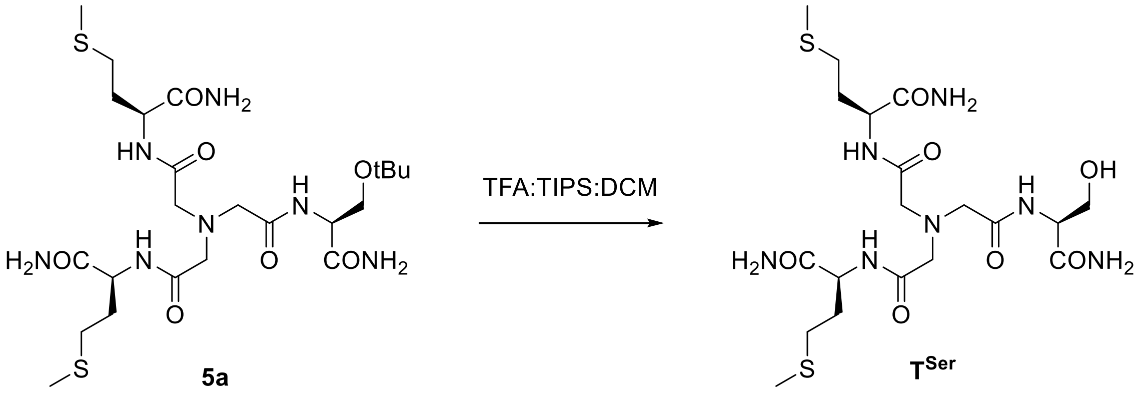

Compound **5a** (0.060 g, 0.101 mmol) was dissolved in DCM (2 mL), followed by addition of TFA (0.54 mL, 7.1 mmol). TIPS (0.04 mL, 0.202 mmol) was added, and the reaction mixture was stirred for 18 h at room temperature. Solvents were evaporated to afford a yellow oil that was purified by preparative reverse-phase HPLC. Gradient: 5% B for 2 min, 5 to 80% B in 10 min and 100% B for 2 min. The desired product was obtained as a white solid (0.051 g, 0.094 mmol, 94%). t_R = 8.41 min (analytical HPLC gradient: 5%B for 2 min, 5 to 100%B in 15 min, 100%B for 2 min).

^{1}H NMR (MeOD, 400 MHz, 298 K): δ (ppm) 4.53-4.45 (m, 3H, $H_{\alpha\text{-Ser}}$, $H_{\alpha\text{-Met}}$), 3.87-3.95 (d, J= 5.56 Hz, 2H, $H_{\beta\text{-Ser}}$), 3.69-3.59 (m, 6H, CH_2-N), 2.65-2.52 (m, 4H, $H_{\gamma\text{-Met}}$), 2.17-2.08 (m, 8H, $H_{\beta\text{-Met}}$, S-CH_3), 2.04-1.95 (m, 2H, $H_{\beta\text{-Met}}$).

^{13}C NMR (MeOD, 125 MHz, 298 K): δ (ppm) 175.17 (NH-C=O), 173.39 (NH_2-C=O_{Ser}), 170.78 (NH_2-C=O_{Met}), 61.60 ($C_{\beta\text{-Ser}}$), 57.58 (CH_2-N), 55.43 ($C_{\alpha\text{-Ser}}$), 52.52 ($C_{\alpha\text{-Met}}$), 31.20 ($C_{\beta\text{-Met}}$), 29.86 ($C_{\gamma\text{-Met}}$), 13.86 (S-CH_3).

ESI-MS (+): M = $C_{19}H_{35}N_7O_7S_2$, m/z calculated for $[M+H]^+$ 538.21, found 538.2.

*Synthesis of **T^MetO***

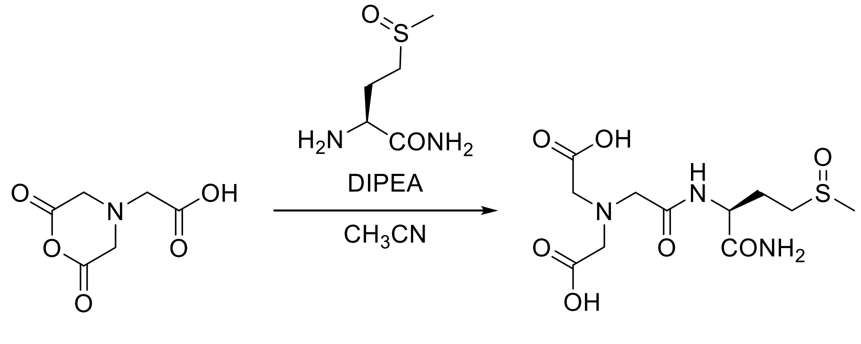

3 **4b**

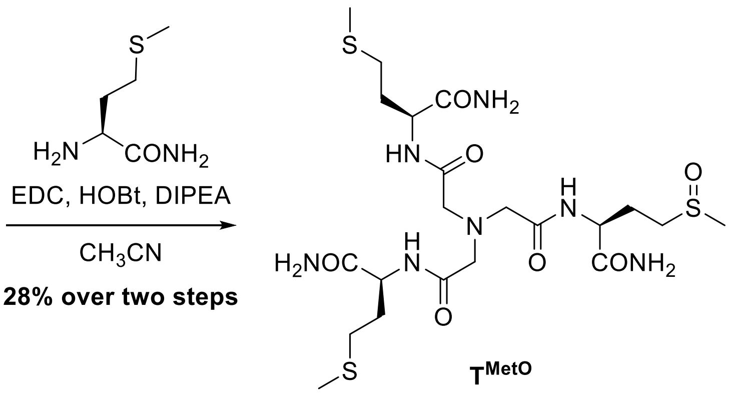

T^MetO

The compound was obtained following general procedure C starting from compound **2b** (0.169 g, 1.030 mmol). HPLC gradient: 15% B for 2 min, 15 to 45% B in 18 min, 45% B for 2 min. The desired product was obtained as a white solid (0.172 g, 0.288 mmol, 28%). t_R = 7.18 min (analytical HPLC gradient: 5%B for 2 min, 5 to 100%B in 15 min, 100%B for 5 min).

^{1}H NMR (MeOD, 400 MHz, 298 K): δ (ppm) 4.59-4.50 (m, 3H, $H_{\alpha\text{-Met}}$, $H_{\alpha\text{-MetO}}$), 3.75-3.53 (m, 6H, CH_2-N), 3.06-2.97 (m, 1H, $H_{\gamma\text{-MetO}}$), 2.92-2.85 (m, 1H, $H_{\gamma\text{-MetO}}$), 2.69 (s, 3H, S(O)-CH_3), 2.66-2.52 (m, 4H, $H_{\gamma\text{-Met}}$), 2.37-2.28 (m, 1H, $H_{\beta\text{-MetO}}$), 2.22-2.09 (m, 9H, $H_{\beta\text{-MetO}}$, $H_{\beta\text{-Met}}$, S-CH_3), 2.04-1.94 (m, 2H, $H_{\beta\text{-Met}}$).

^{13}C NMR (MeOD, 125 MHz, 298 K): δ (ppm) 175.24 (NH_2-C=O_{Met}), 174.21 (NH_2-C=O_{MetO}), 170.95 (N-C=O), 57.68 (CH_2-N_{Met}), 57.21 (CH_2-N_{MetO}), 52.37 ($C_{\alpha\text{-Met}}$, $C_{\alpha\text{-MetO}}$), 49.64 ($C_{\gamma\text{-MetO}}$), 49.37 ($C_{\gamma\text{-MetO}}$), 36.89 (S(O)-CH_3), 31.25 ($C_{\beta\text{-Met}}$), 29.91($C_{\gamma\text{-Met}}$), 25.08 ($C_{\beta\text{-MetO}}$), 24.67($C_{\beta\text{-MetO}}$), 13.89 (S-CH_3).

ESI-MS (+): M = $C_{21}H_{39}N_7O_7S_3$, m/z calculated for $[M+H]^+$ 598.21, found 598.3.

Synthesis of ***T*** $^{Asp(tBu)}$ ***(5c)***

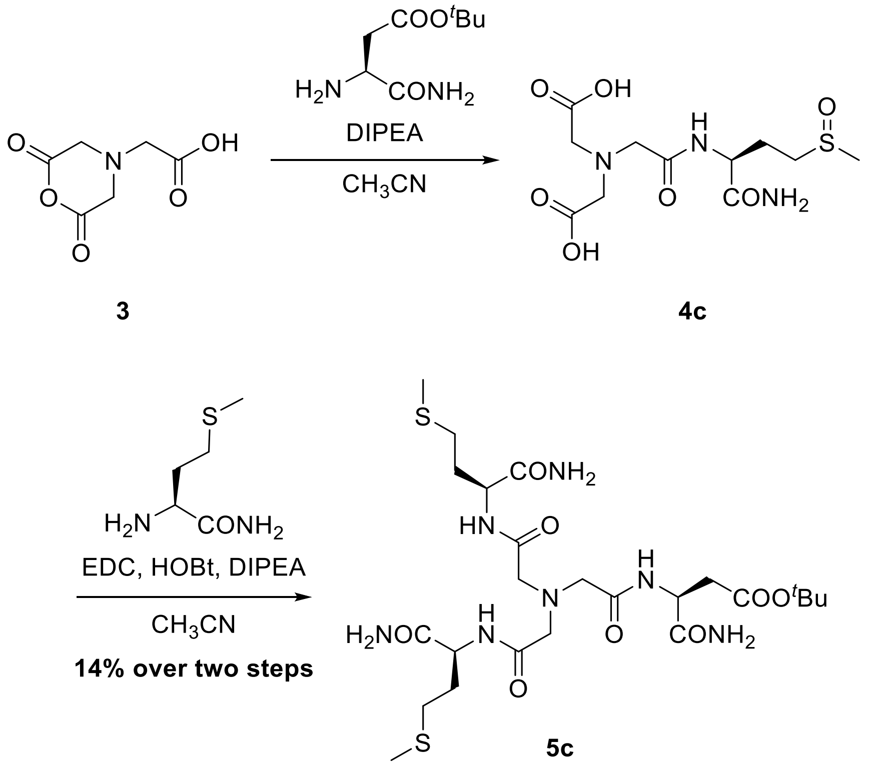

3 **4c** **5c**

The compound was obtained following general procedure C starting from compound **2c** (0.1 g, 0.531 mmol). HPLC gradient: 15% B for 2 min, 15 to 45% B in 18 min, 45% B for 2 min. The desired product was obtained as a white solid (0.0464 g, 0.0746 mmol, 14%). t_R = 10.0 min (analytical HPLC gradient: 15%B for 2 min, 15 to 45%B in 15 min, 45%B for 2 min).

^{1}**H NMR** (MeOD, 400 MHz, 298 K): δ (ppm) 4.78 (dd, J= 13.53, 3.12 Hz, 1H, $H_{\alpha\text{-Asp}}$), 4.51 (dd, J= 14.05, 4.90 Hz, 2H, $H_{\alpha\text{-Met}}$), 3.67-3.52 (m, 6H, CH_2-N), 2.83 (dd, J=5.20, 5.17 Hz, 1H, $H_{\beta\text{-Asp}}$), 2.71-2.52 (m, 5H, $H_{\beta\text{-Asp}}$, $H_{\gamma\text{-Met}}$), 2.18-2.09 (m, 8H, $H_{\beta\text{-Met}}$, S-CH_3), 2.05-1.952 (m, 2H, $H_{\beta\text{-Met}}$), 1.47 (s, 9H, C-CH_3).

^{13}**C NMR** (MeOD, 125 MHz, 298 K): δ (ppm) 175.25 (N-C=O), 174.04 (NH_2-C=O_{Asp}), 170.84 (O-C=O), 170.01 (NH_2-C=O_{Met}), 81.09 (O-C), 57.69 (CH_2-N), 52.38 ($C_{\alpha\text{-Met}}$), 49.68 ($C_{\alpha\text{-Asp}}$), 36.96 ($C_{\beta\text{-Asp}}$), 31.27 ($C_{\beta\text{-Met}}$), 29.90 ($C_{\gamma\text{-Met}}$), 26.94 (CH_3), 13.87 (S-CH_3).

ESI-MS (+): M = $C_{24}H_{43}N_7O_8S_2$, m/z calculated for $[M+Na]^+$ 644.25, found 644.3.

*Synthesis of **T**^Asp^*

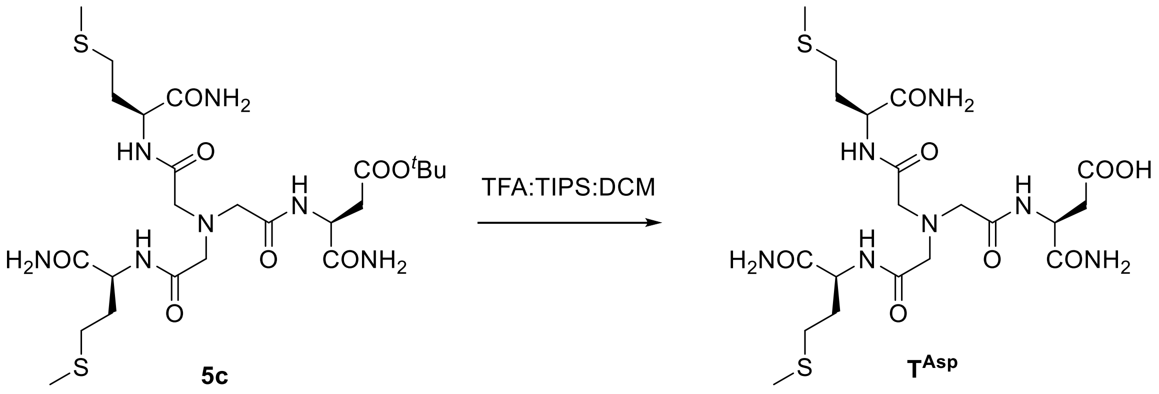

Compound **5c** (0.046g, 0.0740 mmol) was dissolved in 0.5 mL of DCM, followed by the addition of TFA (0.39 mL, 5.183 mmol) and TIPS (0.03 mL, 0.148 mmol). The reaction mixture wasstirred for 18 h at room temperature. Then, solvents were evaporated to yield a yellow oil, that was purified by preparative reverse-phase HPLC. Gradient: 5%B for 2 min, 5 to 80% B in 10 min and 100% B for 2 min. After lyophilization the desired product was obtained as a white solid (0.040 g, 0.071 mmol, 96%). t_R = 6.6 min (analytical HPLC gradient: 5%B for 2 min, 5 to 100%B in 15 min, 100%B for 5 min).

^{1}H NMR (MeOD, 400 MHz, 298 K): δ (ppm) 4.82-4.77 (dd, J= 13.23, 2.93 Hz, 1H, $H_{\alpha\text{-Asp}}$), 4.53-4.49 (dd, J= 14.07, 4.83 Hz, 2H, $H_{\alpha\text{-Met}}$), 3.60-3.47 (m, 6H, CH_2-N), 2.94-2.87 (dd, J= 17.2, 4.8 Hz, 1H, $H_{\beta\text{-Asp}}$), 2.81-2.75 (dd, J= 16.8, 7.6 Hz, 1H, $H_{\beta\text{-Asp}}$), 2.66-2.52 (m, 4H, $H_{\gamma\text{-Met}}$), 2.18-2.01 (m, 8H, $H_{\beta\text{-Met}}$, S-CH_3), 1.95-1.93 (m, 2H, $H_{\beta\text{-Met}}$).

^{13}C NMR (MeOD, 125 MHz, 298 K): δ (ppm) 175.28 (C=O), 174.17 (NH_2-C=O_{Asp}), 172.57 (NH_2-C=O_{Met}), 171.14 (COOH), 57.62 (CH_2-N), 52.39 ($C_{\alpha\text{-Met}}$), 49.66 ($C_{\alpha\text{-Asp}}$), 35.41 ($C_{\beta\text{-Asp}}$), 31.22 ($C_{\beta\text{-Met}}$), 29.90 ($C_{\gamma\text{-Met}}$), 13.87 (S-CH_3).

ESI-MS (-): M = $C_{20}H_{35}N_7O_8S_2$, m/z calculated for $[M-H]^-$ 564.19, found 564.2.

Synthesis of ***T**^{His}*

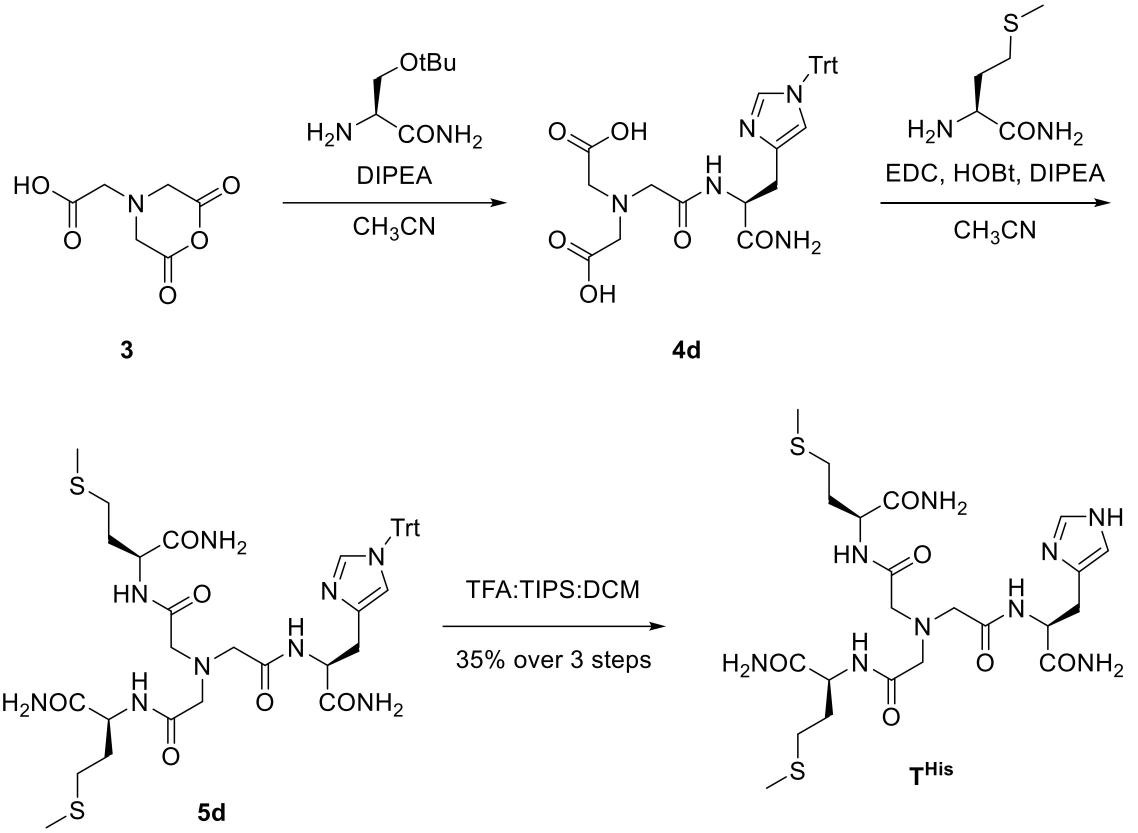

Compound **5d** was obtained using general procedure C starting from compound **2d** (100 mg, 0.252 mmol), and used without HPLC purification for the next step.

The crude mixture was dissolved in DCM (5mL). TFA (0.502 mL, 6.557 mmol) was added to the reaction mixture at 0°C, resulting in a pale yellow solution. TIPS (0.258 mL, 1.261 mmol) was then added dropwise to the solution. After 18 h, solvents were evaporated to obtain a yellow oil that was purified by preparative reverse-phase HPLC. Gradient: 10% B for 2 min, 10 to 20% B in 10 min then 100% B for 2 min. After lyophilisation the desired product was obtained as a white solid (0.052 g, 0.088 mmol, 35%). t_R = 6.98 min (analytical HPLC gradient: 5%B for 2 min, 5 to 100%B in 15 min, 100%B for 5 min).

^{1}H NMR (MeOD, 400 MHz, 298 K): δ (ppm) 8.80 (s, 1H, CH_{ar}), 7.40 (s, 1H, CH_{ar}), 4.75 (dd, J=13.74, 3.90 Hz, 1H, $H_{\alpha\text{-His}}$), 4.51 (dd, J= 14.13, 4.96 Hz, 2H, $H_{\alpha\text{-Met}}$), 3.52-3.37 (m, 7H, CH_2-N, $H_{\beta\text{-His}}$), 3.18 (dd, J=8.87, 8.84 Hz, 1H, $H_{\beta\text{-His}}$), 2.66-2.52 (m, 4H, $H_{\gamma\text{-Met}}$), 2.17-2.04 (m, 8H, S-CH_3, $H_{\beta\text{-Met}}$), 2.02-1.95 (m, 2H, $H_{\beta\text{-Met}}$).

^{13}C NMR (MeOD, 125 MHz, 298 K): δ (ppm) 175.37 (NH-C=O), 173.14 (NH_2-C=O_{His}), 171.60 (NH_2-C=O_{Met}), 133.58 (CH_{ar}), 129.90 (C_{ar}), 117.14 (CH_{ar}), 58.08 (CH_2-N), 57.76 (CH_2-N), 53.35 ($C_{\alpha\text{-Met}}$), 51.93 ($C_{\alpha\text{-His}}$), 31.24 ($C_{\beta\text{-Met}}$), 29.89 ($C_{\gamma\text{-Met}}$), 26.85 ($C_{\beta\text{-His}}$), 13.86 (S-CH_3).

ESI-MS (+): M = $C_{22}H_{37}N_9O_6S_2$, m/z calculated for $[M+H]^+$ 588.24, found 588.3 *m/z*.

Physico-chemical studies.

Ligand solution preparation

Ligands were dissolved in D_2O (ca 5 mM) and aliquots of these solutions were titrated by ^1H NMR using CH_3SO_3Na and CH_3COONa as internal concentration standards. The solutions were then aliquoted and lyophilized to get n aliquot containing a precise number of µmoles of each ligand. This aliquots were then dissolved in water or in the appropriate buffer to get ligand solutions of known concentrations, to run physico-chemical measurements described hereafter. Ligand solutions used for Cu(I) studies were prepared in the glovebox.

Mass spectrometry

Mass spectra were acquired on a LXQ-linear ion trap (THERMO Scientific) instrument equipped with an electrospray ion source. Electrospray full scan spectra have been recorded in the m/z = 50–2000 amu by infusion through a fused silica tubing at a flow rate of 2–10 mL/min. The temperature of the heated capillary for the LXQ was set in the range of 200–250°C, the ion-spray voltage was in the range of 3–6 kV and the injection time was 5–200 ms. Ligand solutions (≈ 200 µM) were prepared in ammonium acetate buffer (20 mM, pH 6.9). Cu(I) was added to the peptide solution from stock solutions of $Cu(CH_3CN)_4PF_6$ in acetonitrile.

UV competition experiments

The apparent affinity constants at pH 7.4 of the Cu(I) complexes were measured by UV-visible titrations in presence of ferrozine (Fz) or ferene (Fs) as a competitor. The spectra were recorded with a Varian Cary50 spectrophotometer equipped with optical fibers connected to an external cell holder in the glove box. The Cu(I) complex was generated from a ligand solution (ca in MOPS buffer (50 mM, pH 7.4) and addition of 0.9 equiv. of $CuSO_4$, in the presence of 2mM hydroxylamine (NH_2OH) and 1 mM ascorbate to *in situ* generate the Cu(I) species.

These solutions were then titrated with Fz or Fs. The spectra were recorded and show the increase of the $Cu(Fz)_2^{3-}$ complex or the $Cu(Fs)_2^{3-}$ complex which absorbs in the visible range: Fz {λ_{max}=470 nm, ε=4 320 cm mol^{-1} L, logβ_{12}=15.1} and Fs {λ_{max}=484 nm, ε=6 700 cm mol^{-1} L, logβ_{12}=13.7}. The stability of the Cu(I)**T** complexes being low, the absorbance measured for 2 equiv. Fs could be used to determine the apparent affinity constants at pH 7.4, in the case of $\mathbf{T^{Met}}$ and $\mathbf{T^{His}}$, only.

Several concentrations of the Cu complex (30- 90 µM) were used to check the reproducibility of the experiments and of the conditional stability constants. A total of 9 experiments were analysed per ligand. GraphPad Prism 10 was used for data representation.

^1H NMR titrations

Ligand solutions in D_2O (ca 2 mM) were titrated by a Cu(I) solution (from 0 to 2 equiv.). The Cu(I) solution was prepared from $CuSO_4$ (40 mM) in D_2O and 4 equiv. of sodium dithionite ($Na_2S_2O_4$). *t*BuOH was added as a non-coordinating, pH-insensitive internal standard for signal chemical shift calibration. Spectra were processed and analysed using Bruker TopSpin 3.6.2. The apparent affinity constants at pH 7.4 of the Cu(I) complexes with the lowest affinity tripods $\mathbf{T^{MetO}}$, $\mathbf{T^{Ser}}$ and $\mathbf{T^{Asp}}$, were measured by ^1H NMR titrations taking $\mathbf{T^{Met}}$ as a reference ligand. Indeed the chemical shift of the SCH_3 group is highly sensitive to Cu(I) coordination and was shown to depend linearly on the fraction of Cu$\mathbf{T^{Met}}$ in the sample thanks to the previous pure ligand titration with Cu(I). Samples containing the ligand of unknown affinity $\mathbf{T^{Aaa}}$, $\mathbf{T^{Met}}$ and Cu(I) prepared from $CuSO_4$ and sodium dithionite, in equimolar concentrations (2 mM) were prepared in D_2O, with *t*BuOH for chemical shift calibration. pDs of all samples were determined after equilibration using a pH-meter (pD = pH* + 0.41), and were within the 6.5-7.2 range. The measurement of $\mathbf{T^{Met}}$ SCH_3 protons chemical shift allowed us to determine [Cu(I)$\mathbf{T^{Met}}$] and [$\mathbf{T^{Met}}$]

in the sample. All the other concentrations could be determined using the value of logβ(Cu^{I}**T**^{Met}) determined though the competition experiment with Fs and the known overall concentrations in the sample.

EPR

The samples for EPR were prepared in acetate buffer (50 mM, pH 5) or MES buffer (50 mM, pH 5.5), with 100 µM solutions of $CuSO_4$ and an excess of the ligands (5-20 equiv.) X-band continuous-wave (CW) EPR measurements were conducted on frozen solutions at 120K under nitrogen gas flow. The spectra were recorded using a Bruker EleXsys E500 spectrometer equipped with a SHQ rectangular cavity and a N_2 temperature controller. All acquisitions were carried out with a microwave power of 10mW and a modulation amplitude of 1.6mT. The reference spectrum with buffer was subtracted from the complex spectra.

Electrochemistry

Electrochemical experiments were performed in a 3-electrode cell comprising a pyrolytic graphite working electrode (PG, surface area 0.071 cm^2), Hg/Hg_2SO_4 reference electrode (sat. K_2SO_4) and a platinum auxiliary electrode. The cell was controlled by Metrohm Autolab PGSTAT101 potentiostat and Nova 2.1 software for data acquisition. Further data analysis was performed in Origin® 2024b software (OriginLab, USA). All measured potentials were converted to the normal hydrogen electrode (NHE) scale by adding 0.62 V to the recorded values. The tripods were dissolved in a MES buffer (pH 5.5, 50 mM) to achieve a final concentration of 1 mM. To eliminate dissolved oxygen, nitrogen was bubbled through the solution prior to adding $CuSO_4$ to reach final concentrations of 20 µM and 50 µM. Cyclic voltammetry (CV) was run before and after $CuSO_4$ addition from 0.7 to - 0.1 V vs NHE at a scan rate 2 mV/s. Square-wave voltammetry (SWV) was run before and after $CuSO_4$ addition from 0.7 to - 0.1 V vs NHE with an amplitude of 20 mV and a frequency of 1 Hz. To test the stability of the complexes towards the oxidation, the CVs were recorded after bubbling the electrochemical cell with oxygen till saturation (1 bar).
The redox potentials of [Cu(II)**T**]/[Cu(I)**T**] were determined from the peak position in the SWV graph. According to the Nernst equation, the peak potential for a reversible redox probe can be linked to the stability constants of the complexes involved:

$$E^{\theta'}_{\mathrm{Cu^{II}T/Cu^{I}T}} = E^{\theta'}_{\mathrm{Cu(II)/Cu(I)}} - \frac{RT}{nF}\ln\frac{\beta_{\mathrm{Cu^{II}T}}}{\beta_{\mathrm{Cu^{I}T}}}$$

where $E^{\theta'}_{\mathrm{Cu(II)/Cu(I)}}$ is the redox potential of free Cu(II)/Cu(I) couple in the aqueous media (0.13 V).[3] It is possible therefore to estimate the stability constant of [Cu(II)**T**] from the redox potential if the stability constant of [Cu(I)**T**] is known:

$$\log\beta_{\mathrm{Cu^{II}T}} = \log\beta_{\mathrm{Cu^{I}T}} - \frac{E^{\theta'}_{\mathrm{Cu^{II}T/Cu^{I}T}} - 0.13V}{0.059V}$$

2. Characterization of the tripodal ligands

^{1}H NMR spectrum of T^{Met} (MeOD, 400 MHz, 298 K)

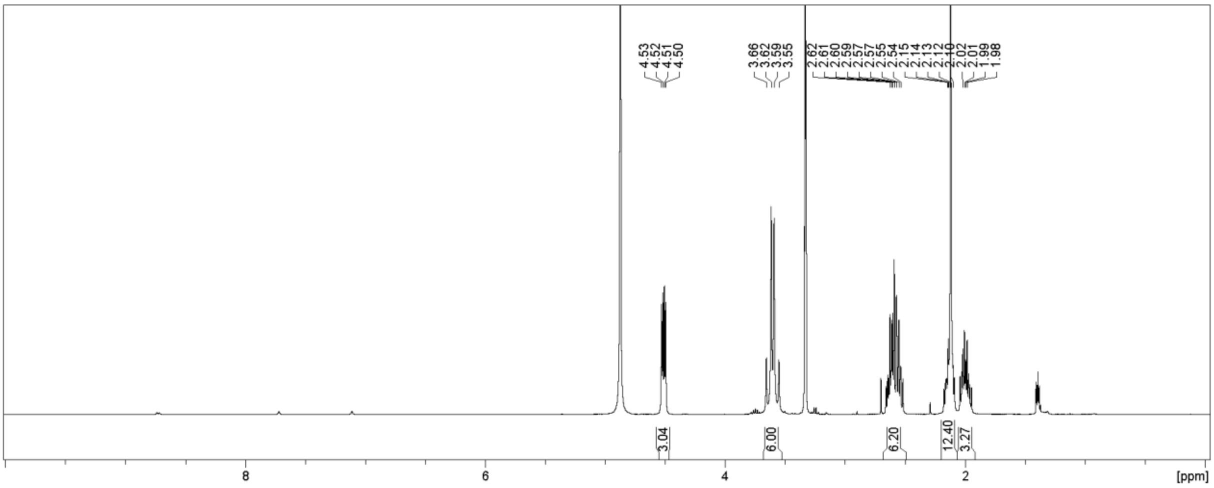

^{13}C NMR spectrum of T^{Met} (MeOD, 125 MHz, 298 K)

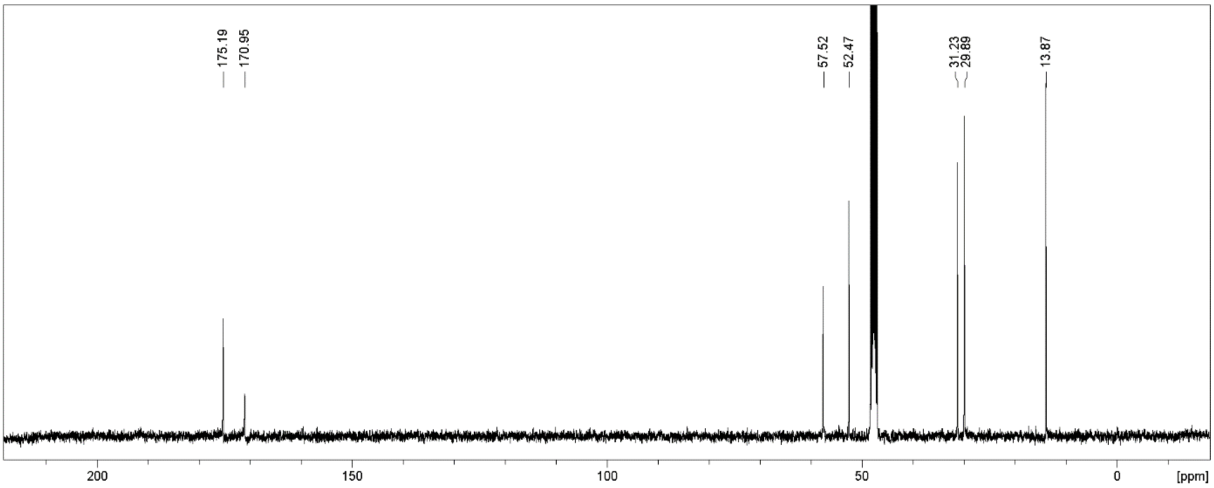

ESI-MS(+) of T^{Met}

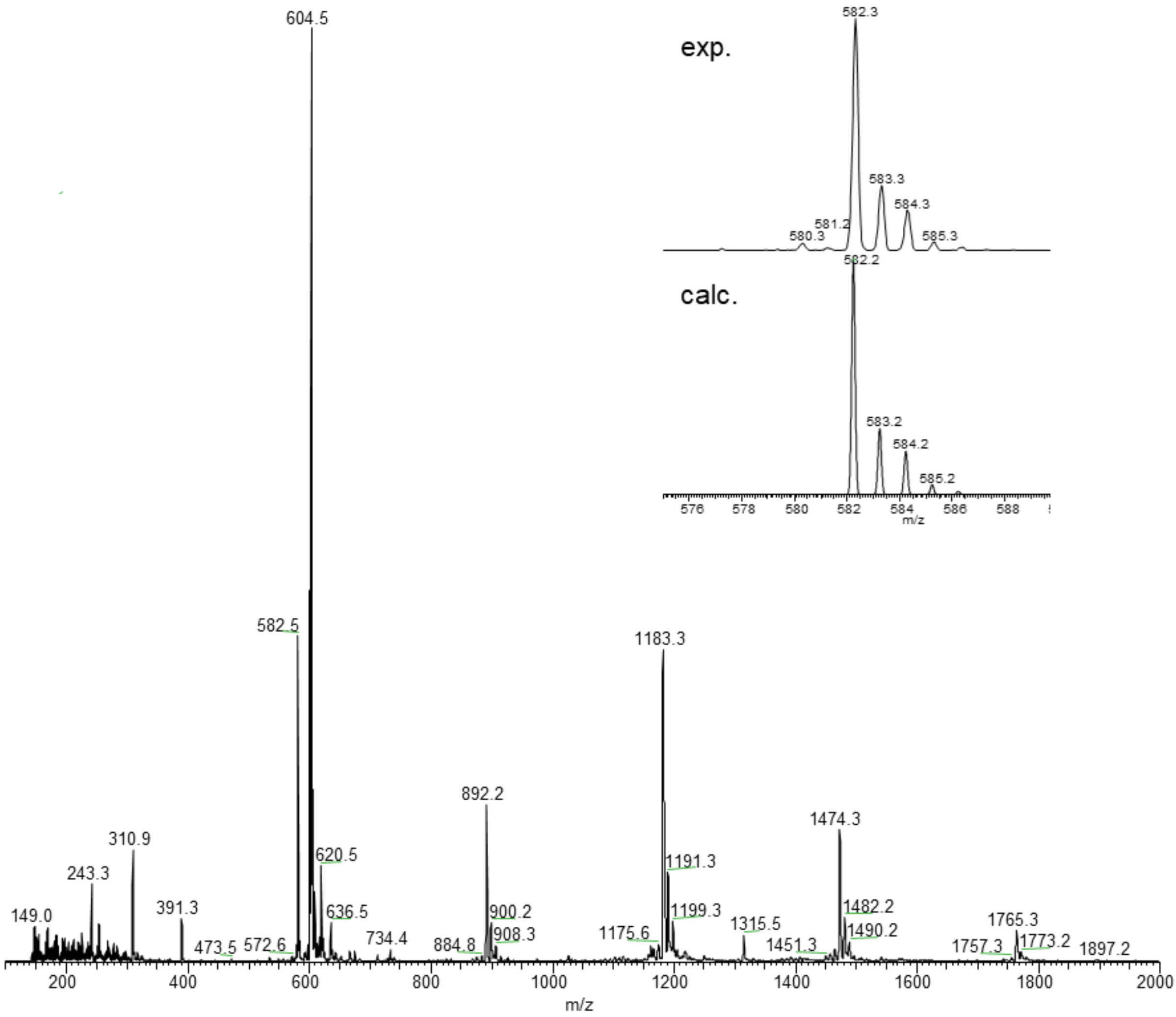

Analytical HPLC of T^{Met}

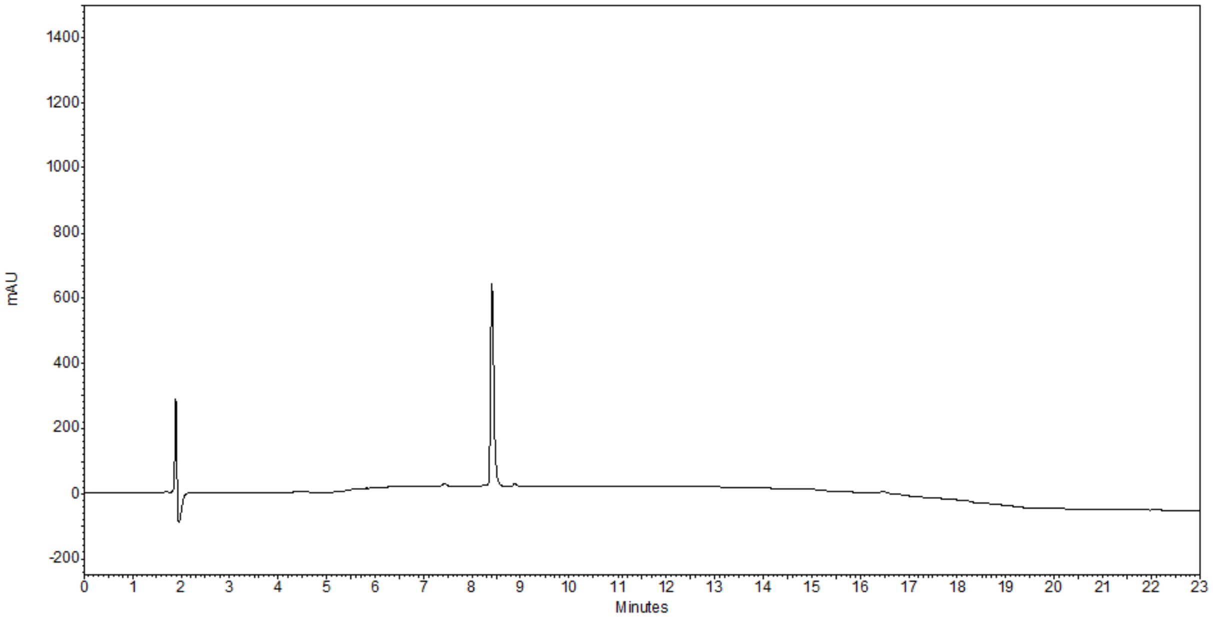

^{1}H NMR spectrum of T^{Ser} (MeOD, 400 MHz, 298 K)

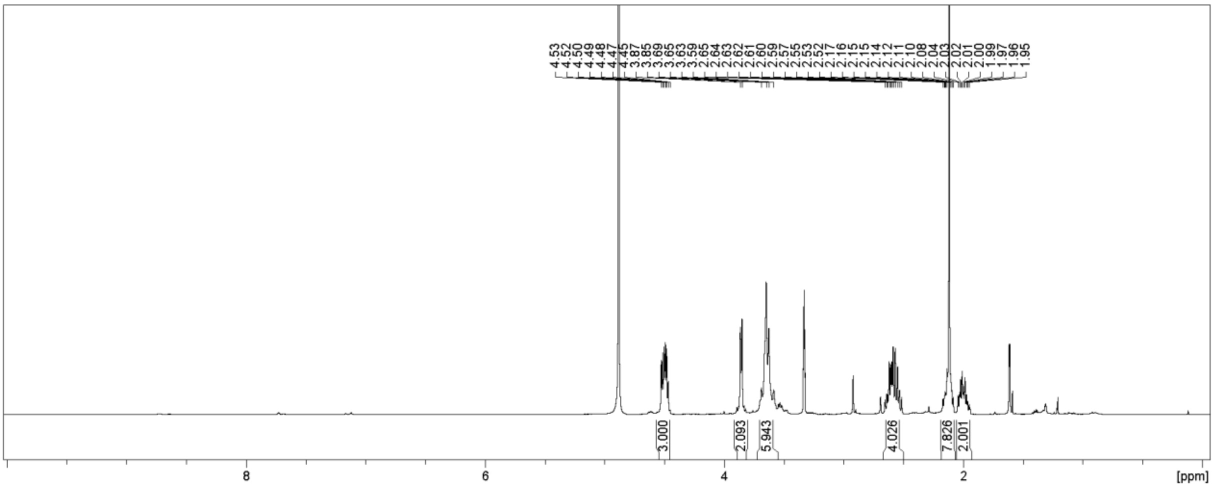

^{13}C NMR spectrum of T^{Ser} (MeOD, 125 MHz, 298 K)

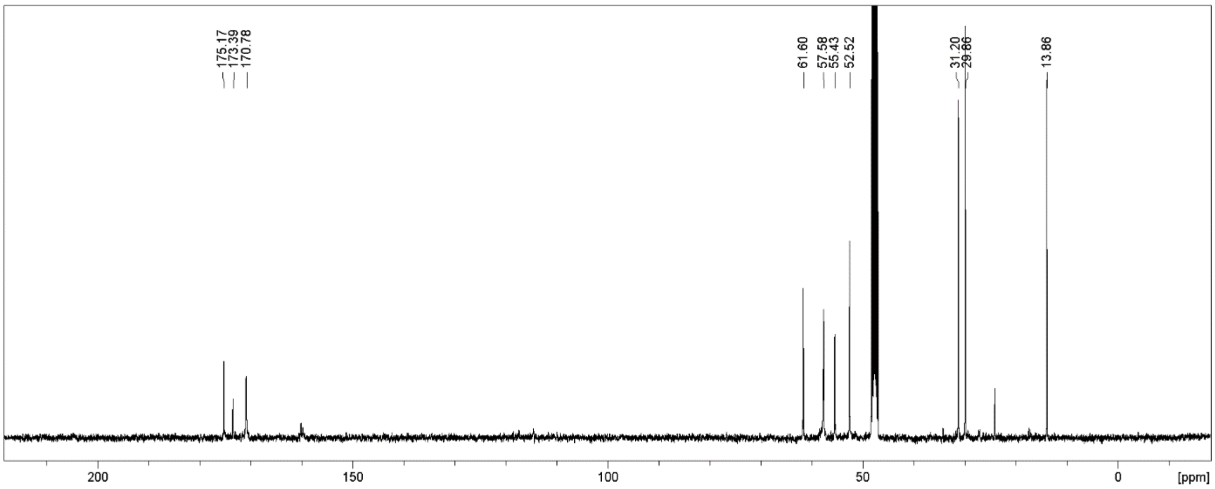

ESI-MS of T^{Ser}

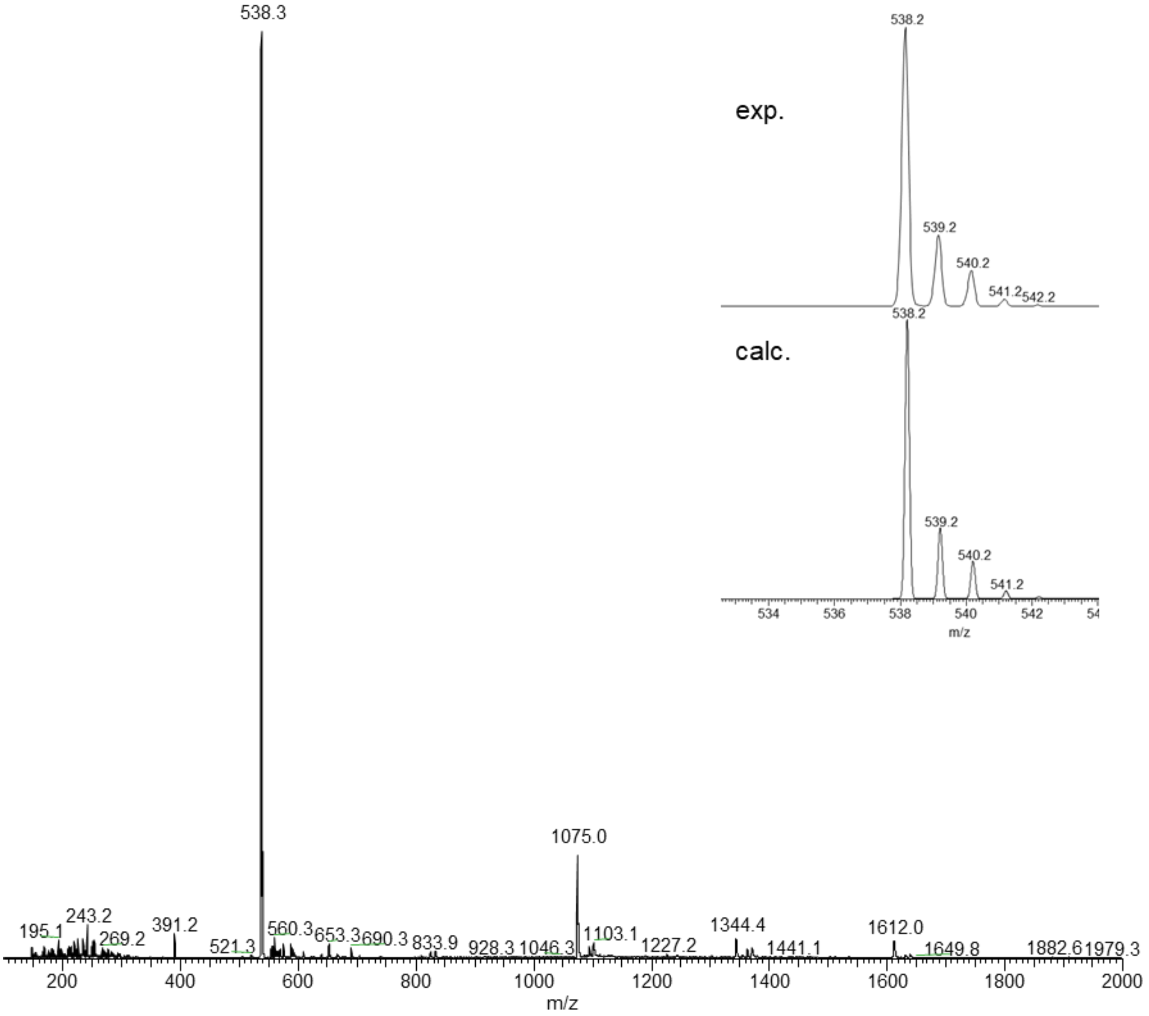

Analytical HPLC of T^{Ser}

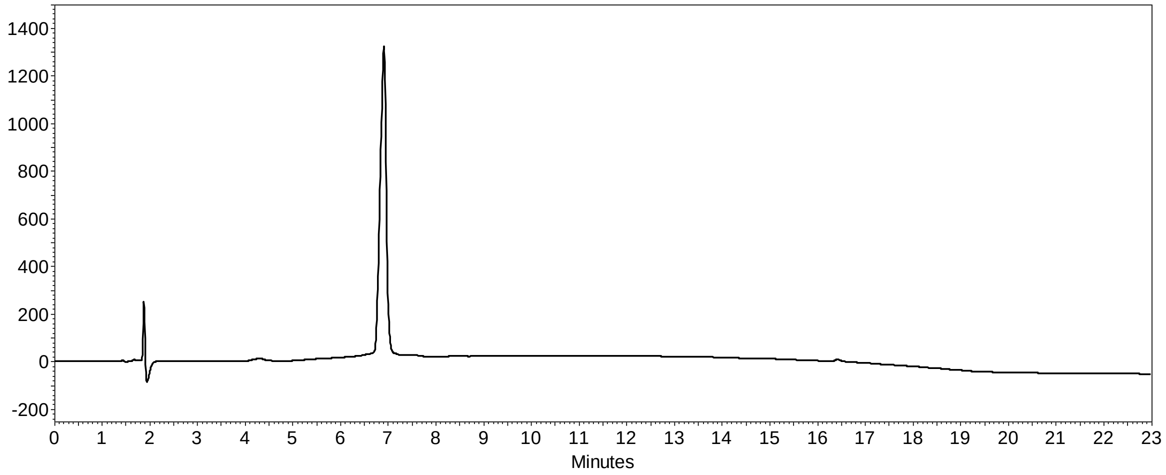

^{1}H NMR spectrum of T^{MetO} (MeOD, 400 MHz, 298 K)

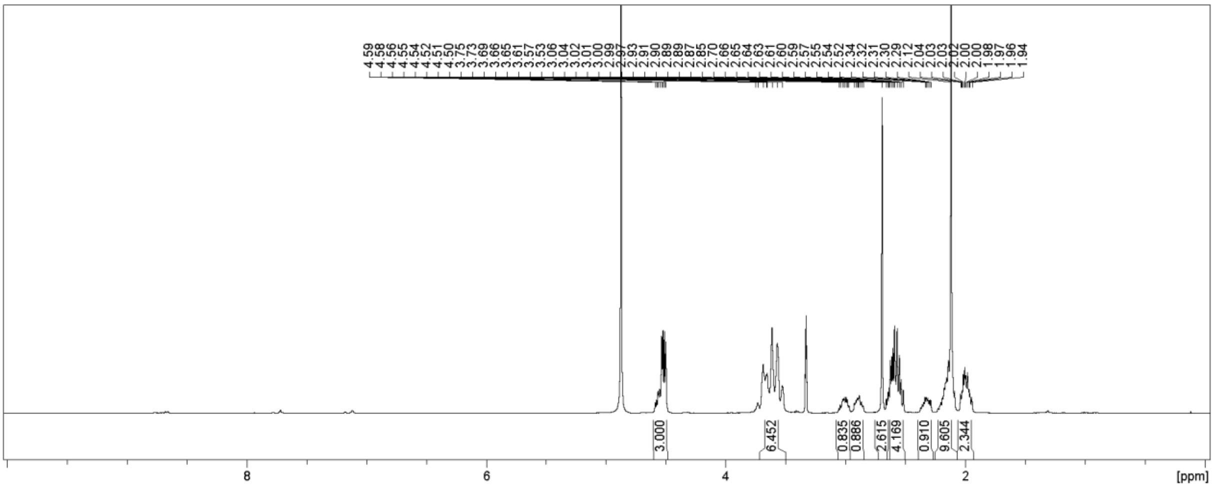

^{13}C NMR spectrum of T^{MetO} (MeOD, 125 MHz, 298 K)

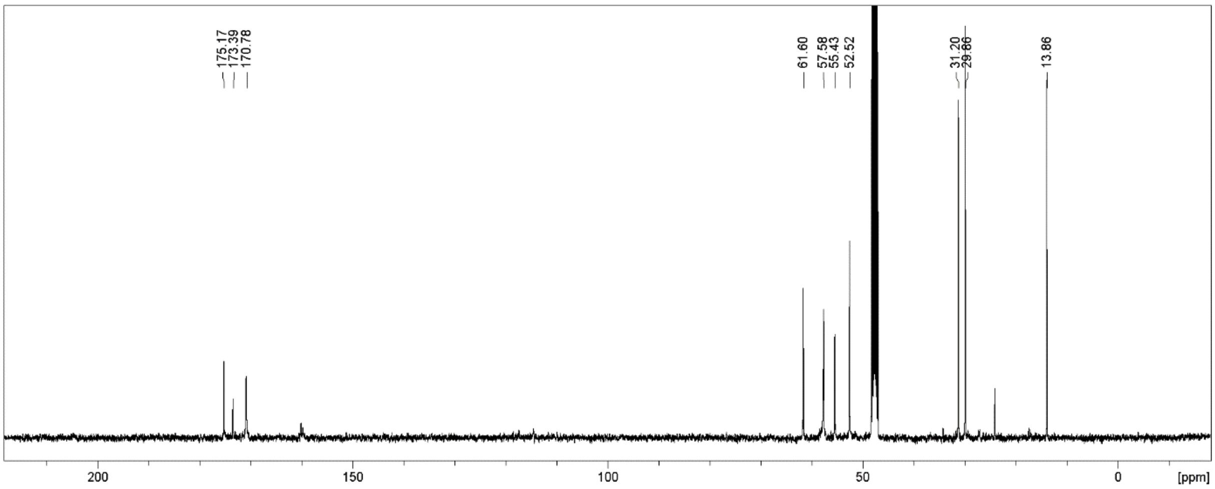

ESI-MS of T^{MetO}

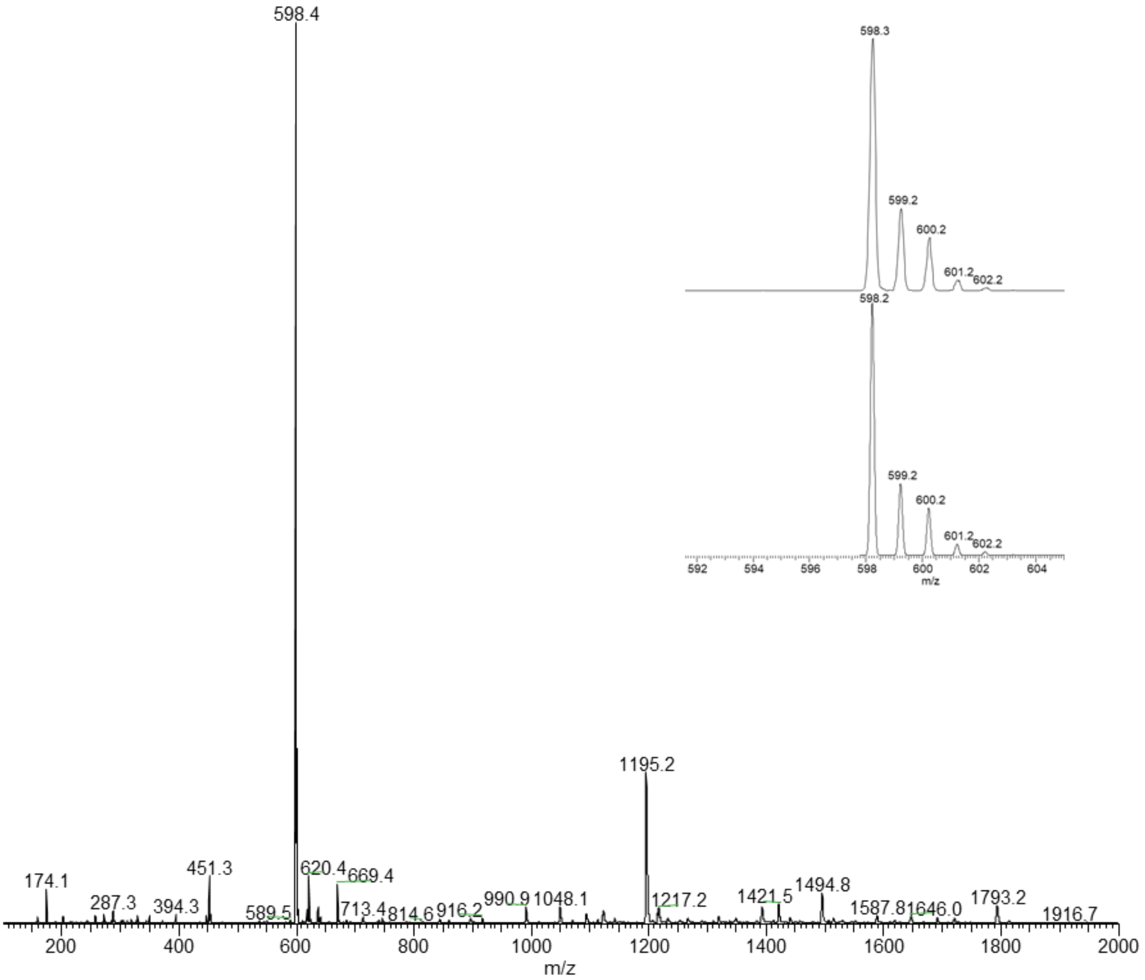

Analytical HPLC of T^{MetO}

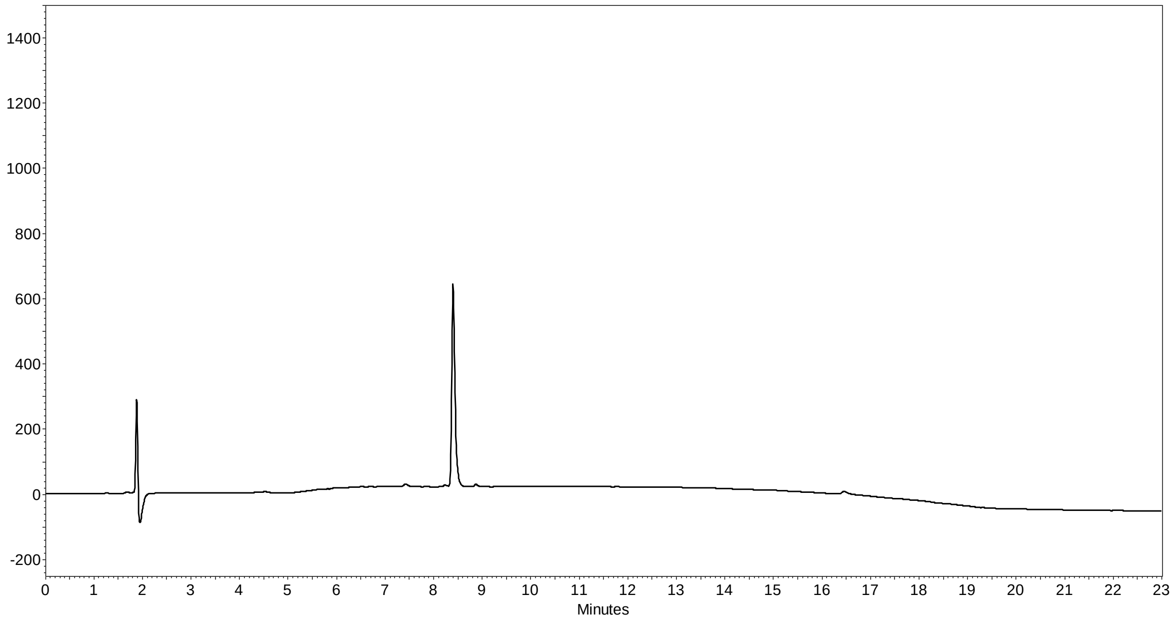

^{1}H NMR spectrum of T[Asp] (MeOD, 400 MHz, 298 K)

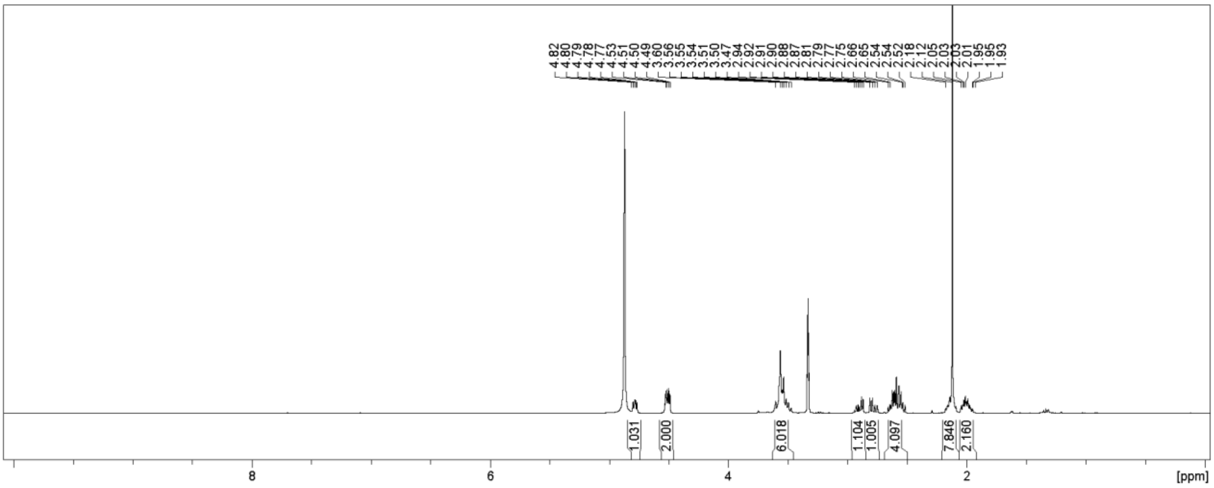

^{13}C NMR spectrum of T[Asp] (MeOD, 125 MHz, 298 K)

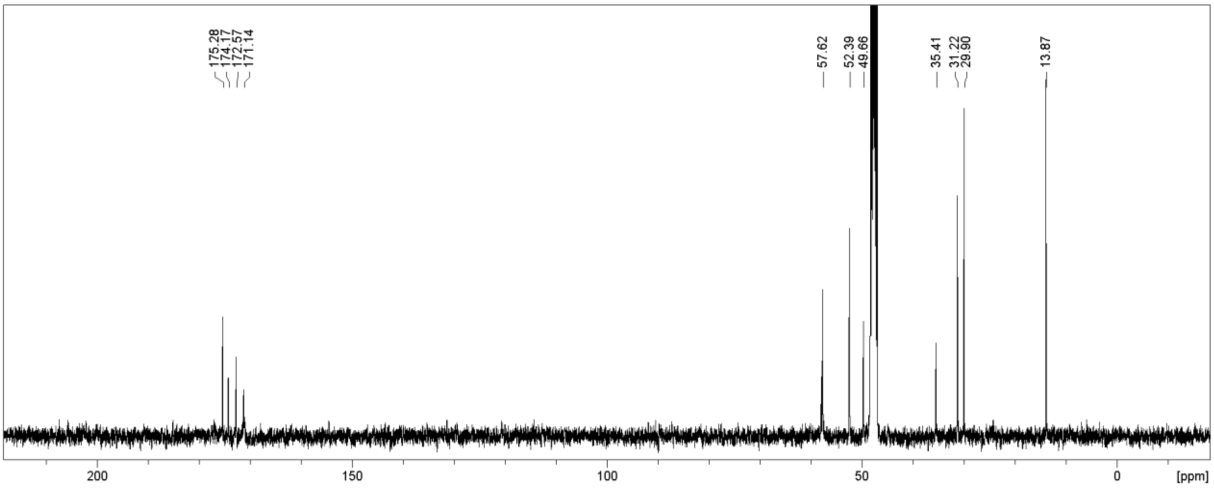

(-)ESI-MS of T^{Asp}

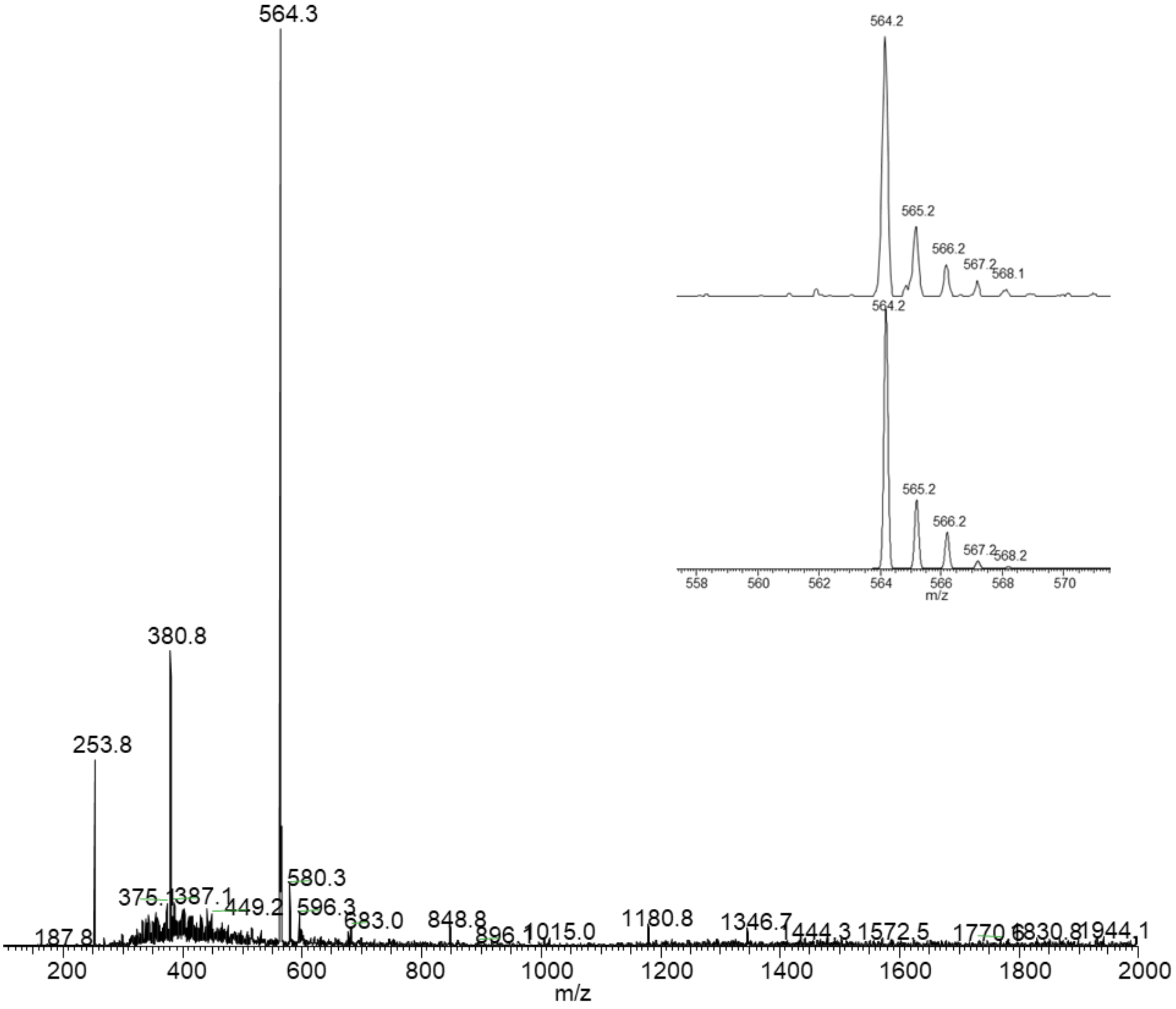

Analytical HPLC of T^{Asp}

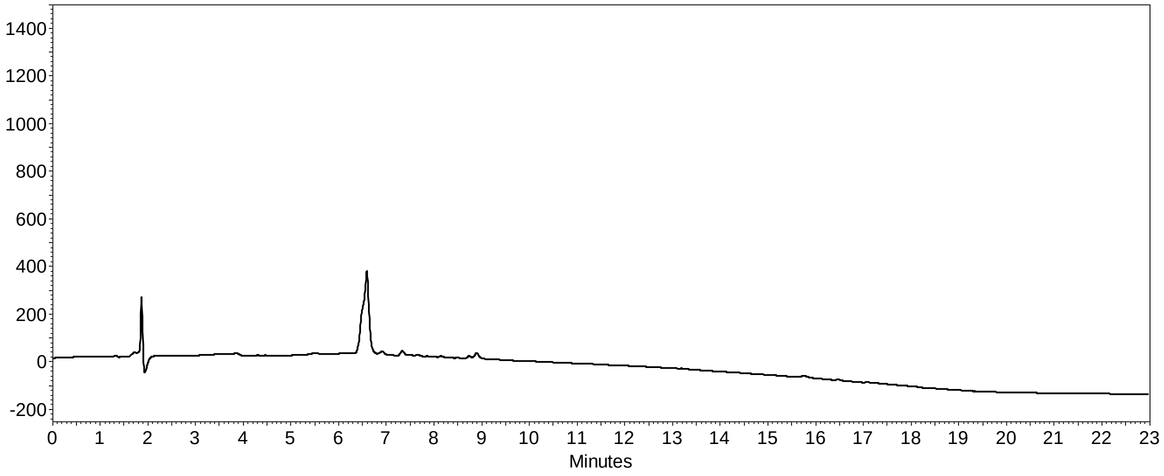

^{1}H NMR spectrum of T^{His} (MeOD, 400 MHz, 298 K):

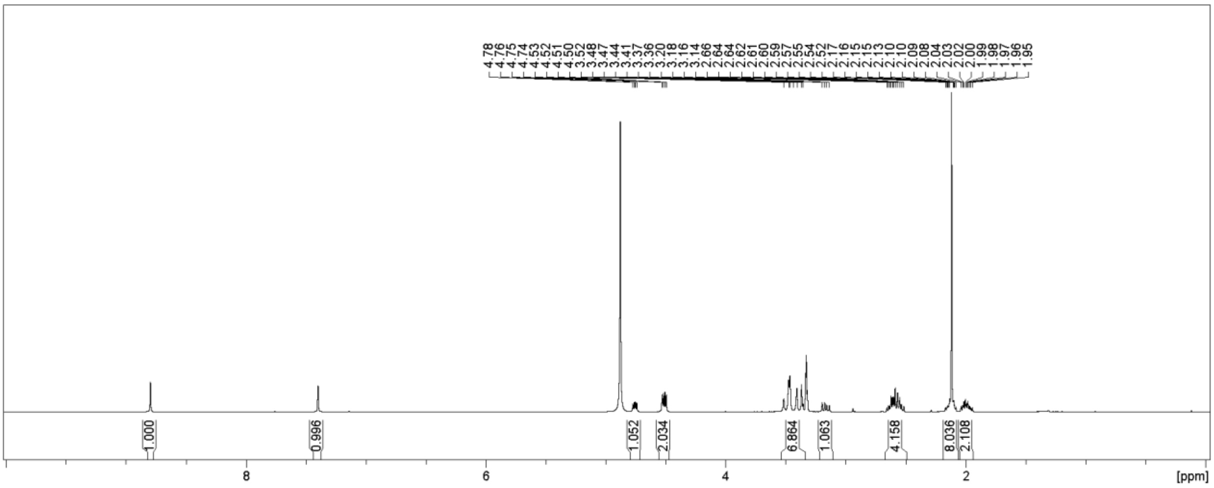

^{13}C NMR spectrum of T^{His} (MeOD, 125 MHz, 298 K)

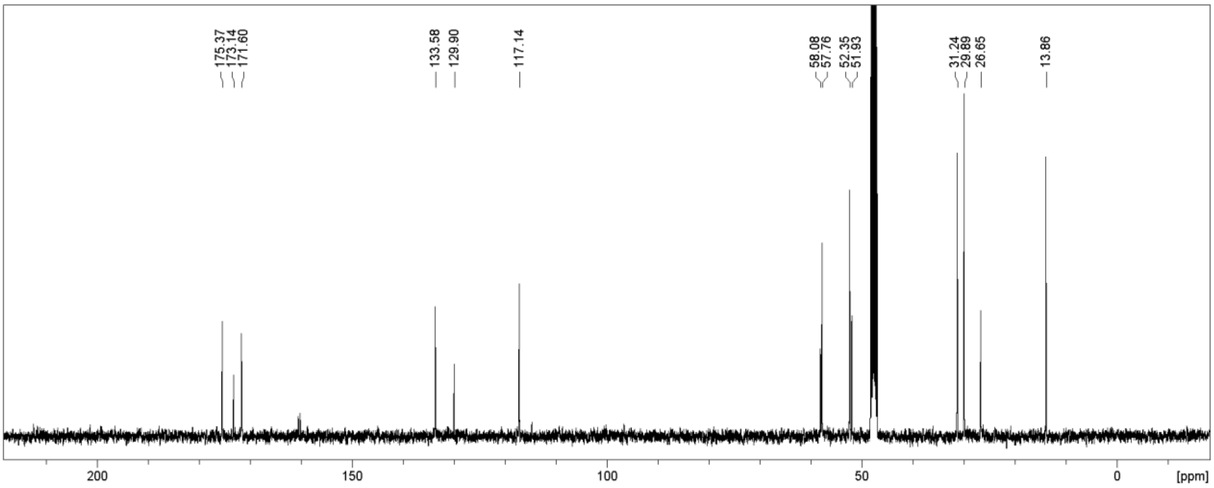

ESI-MS of T^His

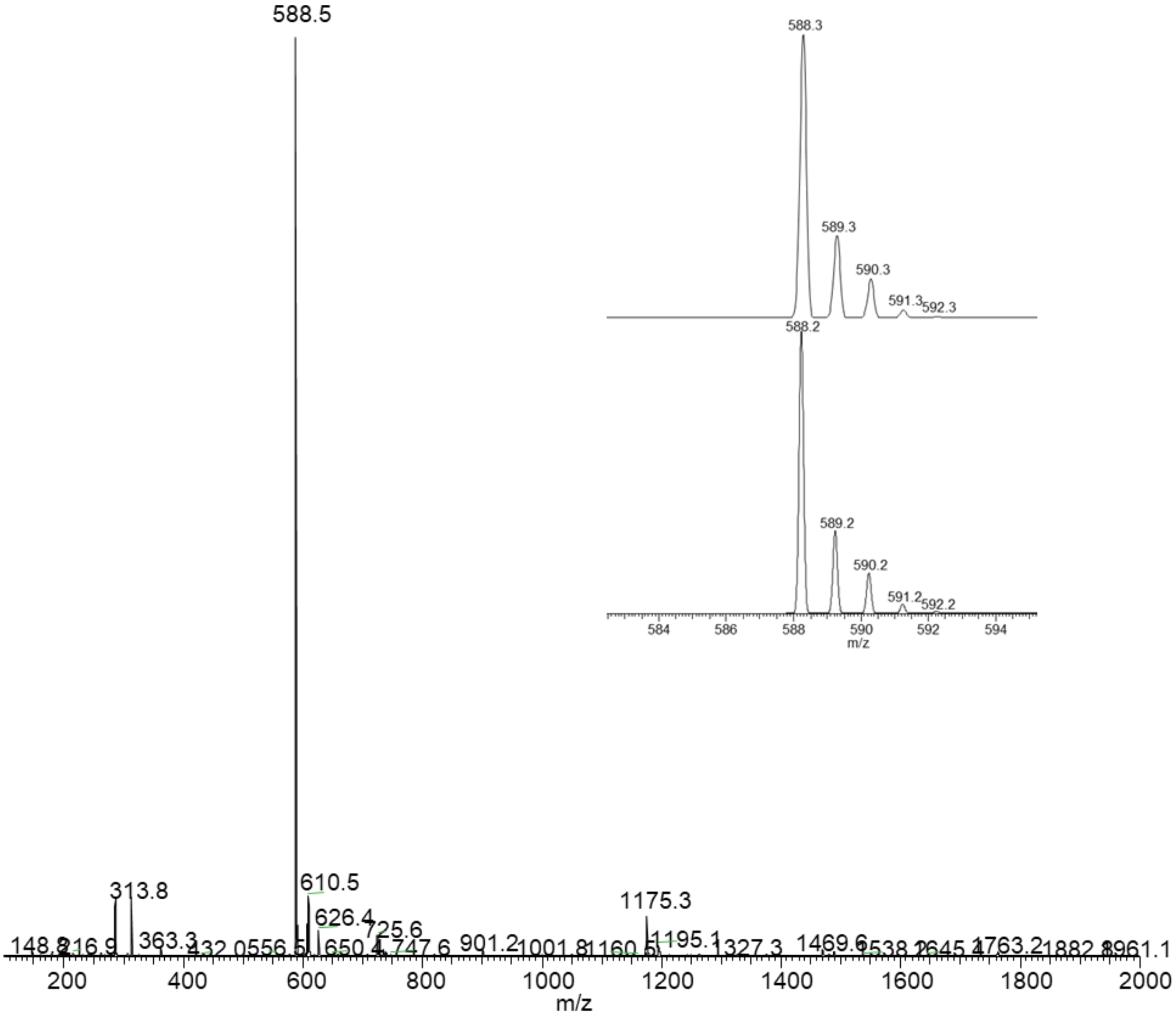

Analytical HPLC of T^His

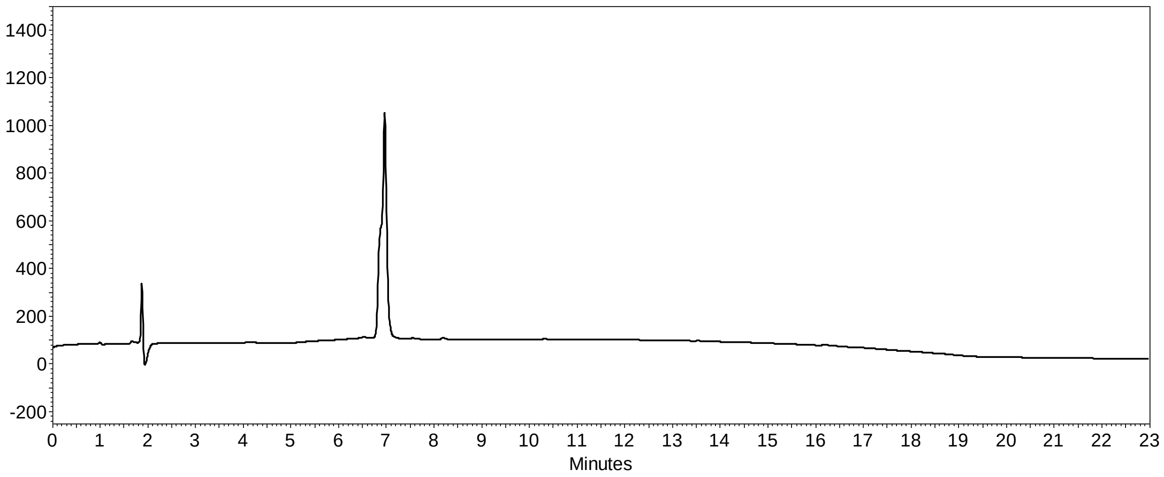

3. Mass spectra of the Cu(I) complexes

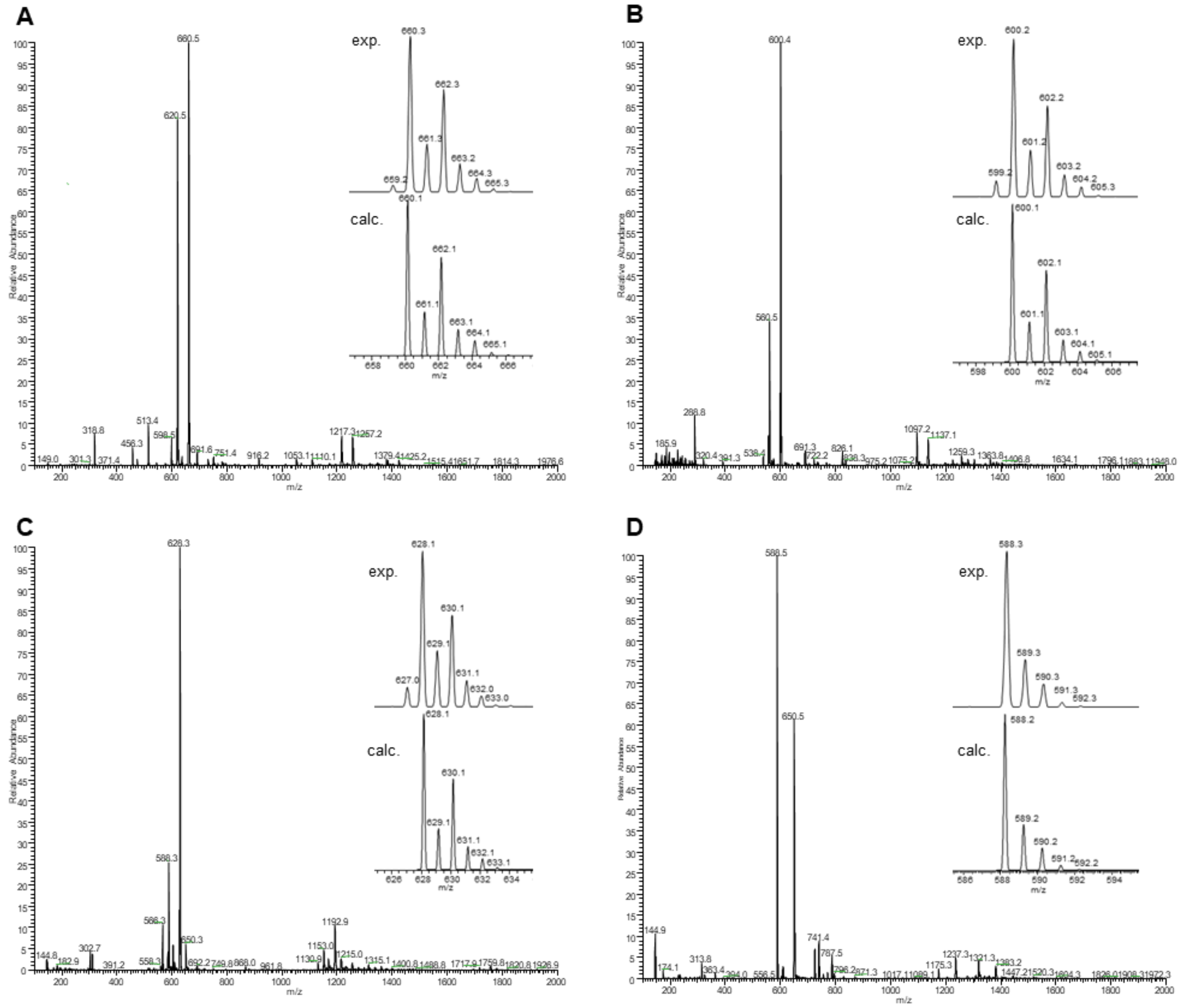

Figure S1. (+)ESI-MS spectra of Cu(I)**T** complexes with A) $\mathbf{T}^{\mathbf{MetO}}$, B) $\mathbf{T}^{\mathbf{Ser}}$, C) $\mathbf{T}^{\mathbf{Asp}}$, D) $\mathbf{T}^{\mathbf{His}}$. (200 µM) in ammonium acetate buffer (20 mM, pH 6.9). Insets show the experimental and calculated isotopic envelops of the [Cu(I)**T**]$^+$ adducts.

4. NMR spectra of the Cu(I) complexes

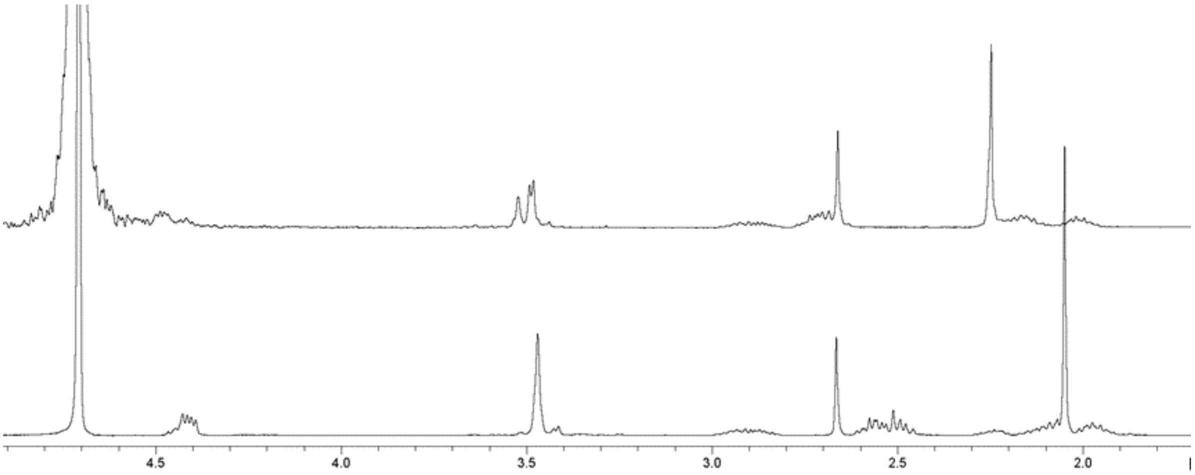

Figure S2. 400 MHz ^{1}H NMR spectra at 298 K in D_2O of $\mathbf{T}^{\mathbf{MetO}}$ (2 mM) with Cu(I): **bottom.** 0 equiv. and **top.** 1 equiv.

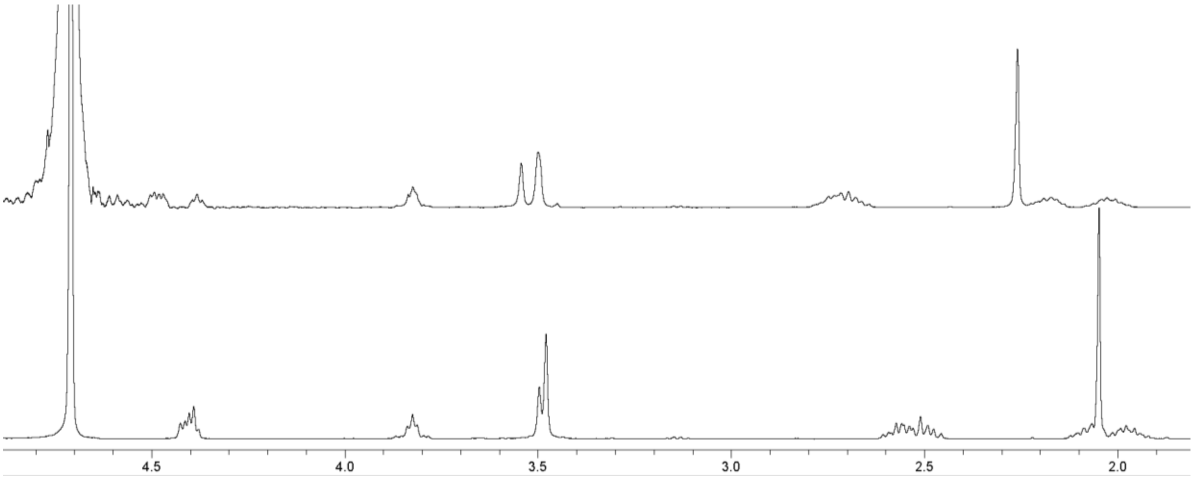

Figure S3. 400 MHz ^{1}H NMR spectra at 298 K in D_2O of $\mathbf{T}^{\mathbf{Ser}}$ (2 mM) with Cu(I): **bottom.** 0 equiv. and **top.** 1 equiv.

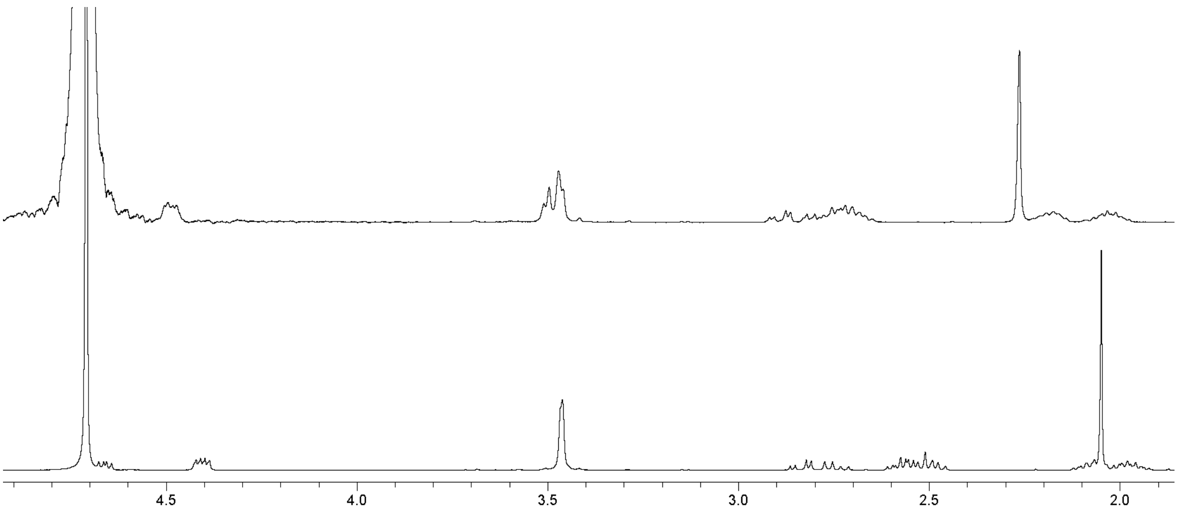

Figure S4. 400 MHz ^{1}H NMR spectra at 298 K in D_2O of $\mathbf{T^{Asp}}$ (2 mM) with Cu(I): **bottom.** 0 equiv. and **top.** 1 equiv.

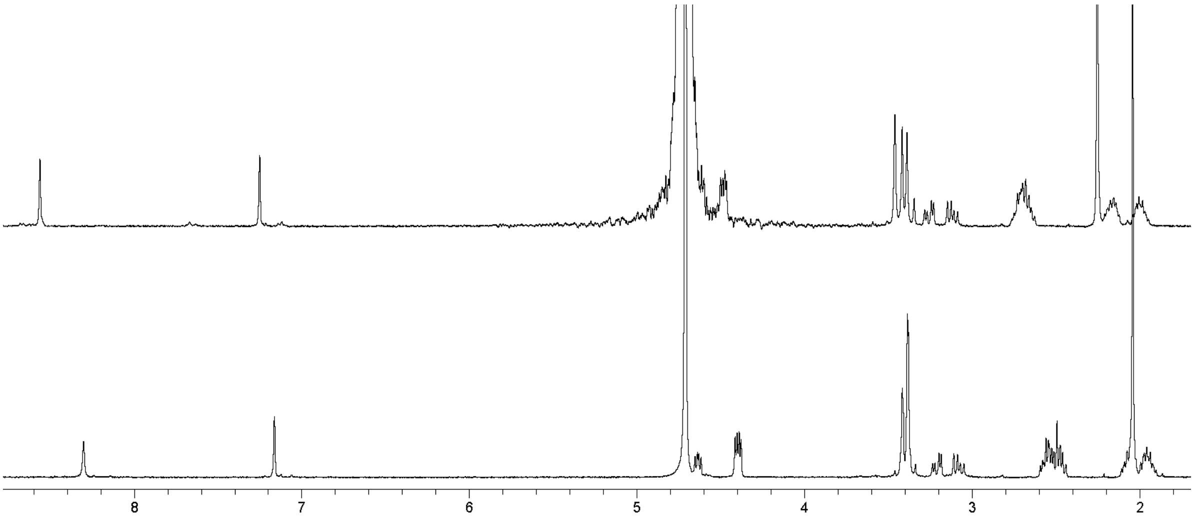

Figure S5. 400 MHz ^{1}H NMR spectra at 298 K in D_2O of $\mathbf{T^{His}}$ (2 mM) with Cu(I): **bottom.** 0 equiv. and **top.** 1 equiv.

5. Competition experiments

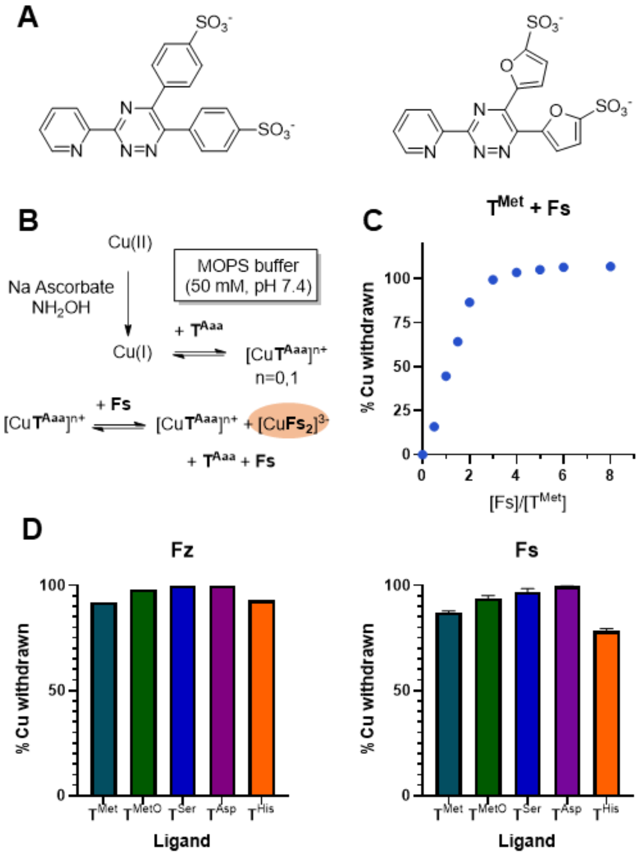

Figure S6. UV competition experiments. (A) structure of Ferrozine^{TM} (Fz, left) and Ferrene^{TM} (Fs, right). (B) Principle of the competition assay in the case of Fs. The absorbance of $[Cu\mathbf{Fs}_2]^{3-}$ is monitored at 484 nm. (C) %Cu withdrawn from $[Cu\mathbf{T}^{\mathbf{Met}}]^+$ as a function of $[Fs]/[\mathbf{T}^{\mathbf{Met}}]$. (D) %Cu withdrawn from $[Cu\mathbf{T}^{\mathbf{Aaa}}]^+$ at 2 equiv. **Fz** (left) or **Fs** (right).

6. EPR spectra of the Cu(II) complexes

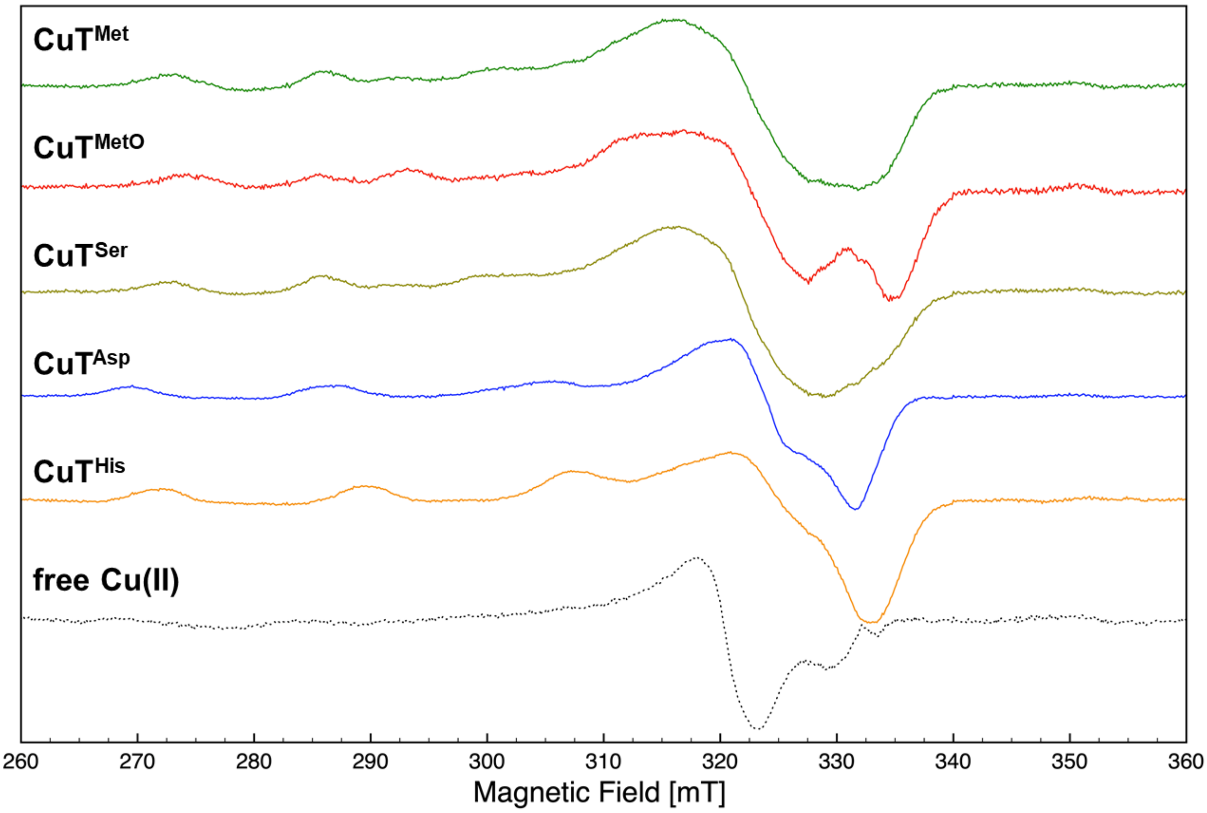

Figure S7. EPR spectra of Cu(II):tripod complexes. 100 µM Cu(II) solutions in 50 mM MES buffer (pH 5.5) were prepared in the presence of 2 mM tripod ligand (20 equiv.). Acquisition parameters: ν = 9.4 GHz, T = 120K, P = 10 mW, MA=1.6mT (100 kHz).

Table S1. Estimated g_z values and hyperfine coupling constants A_z^{Cu} for the different species detected in Cu(II) complexes.

		g_z	A_z^{Cu} [MHz]
**T^{Met}**	major species	2.28(1)	425(10)
	minor species	2.21(1)	542(10)
**T^{MetO}**	major species	2.21(1)	542(10)
	minor species	2.28(1)	425(10)
**T^{Ser}**	major species	2.28(1)	425(10)
	minor species	2.21(1)	542(10)
**T^{Asp}**	major species	2.25(1)	567(10)
	minor species	2.27(1)	509(10)
**T^{His}**		2.23(1)	556(10)

7. Electrochemistry of the Cu complexes

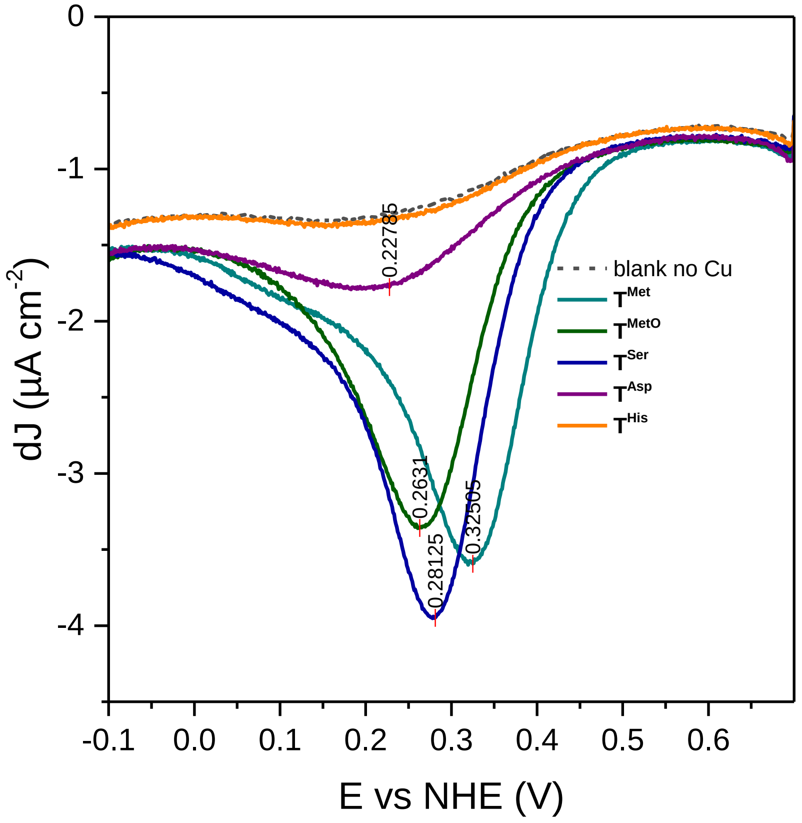

Figure S8. Square-wave voltammograms (SWV) of Cu(II):tripod complexes. SWV experiments were performed at RT under N_2, on 50 µM Cu(II) solutions in 50 mM MES buffer (pH 5.5), in the presence of 1 mM tripod ligand (20 equiv.). Amplitude 20 mV, frequency 1 Hz.

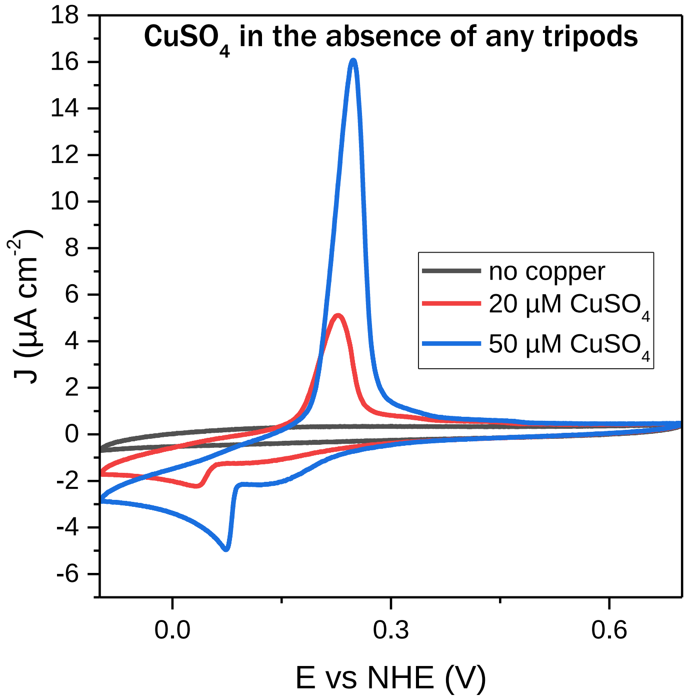

Figure S9. CVs of $CuSO_4$ solutions in MES buffer (50 mM, pH 5.5) in the absence of tripod pseudopeptides at RT under N_2.

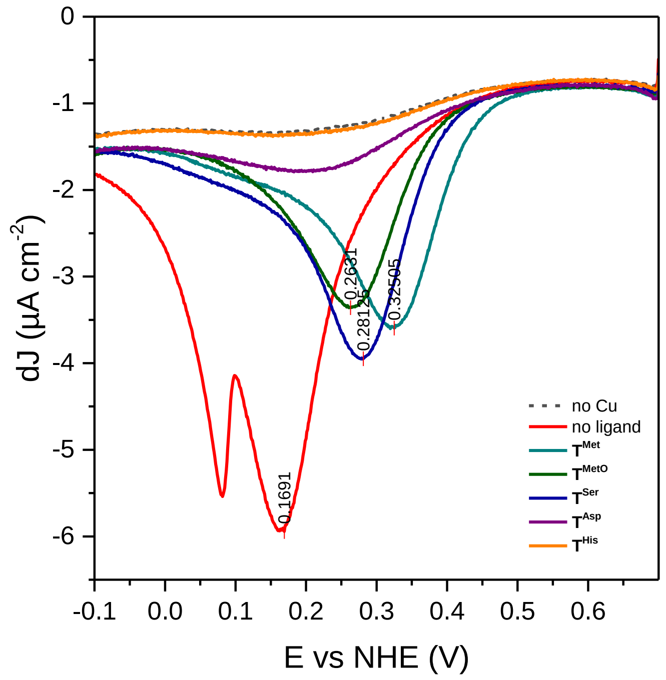

Figure S10. Square-wave voltammetry (SWV) of Cu(II):tripod complexes compared to $CuSO_4$ (50 µM in MES buffer pH 5.5).

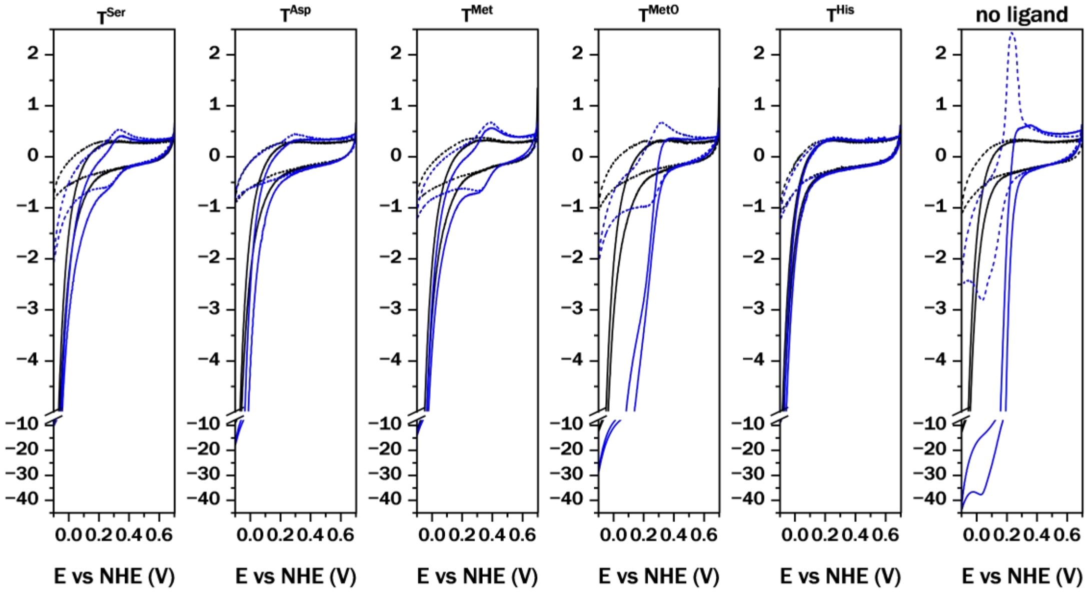

Figure S11. CVs of solutions of **T^Ser^**, **T^Asp^**, **T^MetO^**, **T^Met^** and **T^His^** (1 mM) in MES buffer (50 mM, pH 5.5) under N_2 (dashed curves) and O_2 (solid curves). Scan rate: 2 mV s^{-1}. [$CuSO_4$] = 0 (black curves), 20 µM (blue curves).

8. References

[1] A.-S. Jullien, C. Gateau, C. Lebrun, P. Delangle, *Inorg. Chem.* **2015**, *54*, 2339–2344.

[2] A. M. Attar, M. B. Richardson, G. Speciale, S. Majumdar, R. P. Dyer, E. C. Sanders, R. M. Penner, G. A. Weiss, *ACS Appl. Mater. Interfaces* **2019**, *11*, 4757–4765.

[3] E. A. Ambundo, M.-V. Deydier, A. J. Grall, N. Aguera-Vega, L. T. Dressel, T. H. Cooper, M. J. Heeg, L. A. Ochrymowycz, D. B. Rorabacher, *Inorg. Chem.* **1999**, *38*, 4233–4242.
